# Relative Leukocyte Telomere Length Is Shorter in Children and Adolescents with Type 1 Diabetes: Screening of Basic Psychosocial Aspects

**DOI:** 10.3390/ijms27093895

**Published:** 2026-04-27

**Authors:** Georgia Papavasileiou, Eleni Dragona, Nicolas C. Nicolaides, Tania Siahanidou, Maria Michou, Emmanouil Zoumakis, Sarantis Gagos, Christina Kanaka-Gantenbein

**Affiliations:** 1 Post-Graduate Course of Stress Science and Health Promotion, Medical School, National and Kapodistrian University of Athens, 11527 Athens, Greece; georgiapapav@med.uoa.gr (G.P.); nnicolaides@bioacademy.gr (N.C.N.); siahan@med.uoa.gr (T.S.); mariamixou@hotmail.com (M.M.); mzoumak@med.uoa.gr (E.Z.); 2 Laboratory of Genetics, Centre of Experimental Medicine and Translational Research, Biomedical Research Foundation of the Academy of Athens, 11527 Athens, Greece; edragona@bioacademy.gr (E.D.); sgagos@bioacademy.gr (S.G.); 3 Division of Endocrinology, Metabolism and Diabetes, First Department of Pediatrics, Medical School, National and Kapodistrian University of Athens, Aghia Sophia Children’s Hospital, 11527 Athens, Greece; 4 Neonatal Unit, First Department of Pediatrics, Medical School, National and Kapodistrian University of Athens, Aghia Sophia Children’s Hospital, 11527 Athens, Greece

**Keywords:** leukocyte telomere length, type 1 diabetes, children, perceived social support, stress

## Abstract

Leukocyte telomere length (LTL) is shortened in adults with type 1 diabetes (T1D), but less data is available concerning pediatric cases. Multiple factors affect LTL, namely genes, epigenetics, environmental factors, oxidation, and psychological stress. Children with T1D and their families experience chronic stress. This study aimed to investigate LTL in children with T1D (n = 35) aged 6–13 years old, in comparison to age-matched healthy counterparts (n = 35), and assess any correlation of LTL with perceived stress. Relative LTL (rLTL) was assessed through real-time qPCR. Morning serum cortisol, high-sensitivity C-Reactive Protein (hsCRP), and glycated hemoglobin (HbA1c) were measured. Children completed the validated questionnaires “Stress in Children” and “Pediatric Quality of Life”. Parents answered the “Perceived Stress Scale”. Children with T1D had a lower rLTL (*p* = 0.02) compared to age-matched healthy controls, higher hsCRP (*p* = 0.031), and a lower estimated quality of life (*p* = 0.01). RLTL was found to be lower in females with T1D (*p* < 0.001) and was positively related to the ‘gender–social support’ factor (*p* = 0.002) and diabetes duration (*p* = 0.045), adjusted for children’s age, parental age, and sociodemographic characteristics. These pilot findings indicate early emergence of shorter rLTL in T1D, pointing to a sexual dimorphism pattern. Insights into preventing LTL shortening in pediatric T1D can be gained from large-scale studies examining the impact of gender and social support.

## 1. Introduction

Type 1 diabetes mellitus, otherwise mentioned as type 1 diabetes (T1D), is a chronic disease leading to progressive autoimmune destruction of the insulin-producing pancreatic β-cells in genetically predisposed subjects [1]. Characterized by absolute insulin deficiency, T1D is the most common type of diabetes in children and adolescents [2] and presents a worldwide annual increase of new cases of around 2–5% [3]. Complications arise from inadequate glycemic control and can be both acute, such as diabetic ketoacidosis (DKA), hypoglycemia, and hyperosmolar hyperglycemic state, and chronic, including diabetic nephropathy, retinopathy, and polyneuropathy [1]. Chronic hyperglycemia leads to the formation of advanced glycated end products (AGEs), even in young children [4]. Accumulation of AGEs in diverse cell types exerts alterations in the structure of the extracellular membrane, dysfunction of intracellular proteins and membrane receptors, triggering inflammatory processes that provoke injury to the vascular endothelium integrity, resulting in progressive accelerated cell aging [5]. Understanding the molecular mechanisms that underlie T1D pathophysiology may help prevent diabetes complications and improve disease management.

One mechanism related to cumulative cellular senescence in humans is telomere attrition [6]. Telomeres are non-coding TTAGGG repeated sequences, capping the ends of eukaryotic chromosomes. They preserve the integrity and stability of coding DNA and sustain cell homeostasis [7]. Several factors affect their initial length, such as heredity [7,8] and epigenetics [8,9]. The genetically determined percentage of LTL highly ranges between 36% and 86% [9]. Telomeres naturally shorten over time during cellular replication until they reach a critical length but undergo accelerated shortening under conditions of high cellular stress and inflammation [10], such as oxidative stress [11]. Telomerase is the enzyme that serves to maintain telomeres [6] but is absent in most human somatic cells after birth, whereas in leukocytes, telomerase activity is retained [6]. Decreased leukocyte telomere length (LTL) is associated with cell aging and has been predictive of greater disease burden [12], although LTL is not yet established as a specific biomarker.

Research interest regarding telomeres in patients with diabetes mellitus (DM) emerged in 1998 by Jeanclos and colleagues [13]. This and subsequent research [14,15,16] reported a shorter peripheral TL (leukocyte and whole blood sample) in adult patients with T1D compared to healthy adults. Biological mechanisms linking telomere attrition with existence of T1D include hyperglycemia, increased glucose variation, obesity, diabetic ketoacidosis [17], nitrosative stress [18], and dysregulated cellular homeostasis [19]. Nevertheless, the severity of hyperglycemia in T1D is not conclusively related to emergence of shorter LTL in longitudinal [18,20] and cross-sectional studies [13,16], nor with oxidation status [15,16], diabetes duration [16,18], or disease complications [20,21]. Researchers speculate that beyond heredity, unknown pathophysiological mechanisms interfere with the function and expression of telomeres in T1D [13,22]. Conversely, shorter telomeres have been hypothesized to induce a highly inflammatory environment leading to cellular dysfunction [7]. Recently, a publication utilizing Mendelian randomization analyses presented the complex bidirectional relationship between TL and T1D, where insulin therapy showed a compensatory effect on LTL shortening [19]. Scarce data concerning LTL in children with T1D point out the importance of euglycemia in preventing telomere attrition [18]. Telomere dynamics in children, growth hormone fluctuations, and immune system maturation could also interrelate to telomere measurements through processes that demand clarification [9].

The impact of perceived psychological stress on telomere attrition was initially examined by Epel and colleagues [23]. Chronic psychological distress [23], adverse childhood experiences, and low social support [24] have been associated with shorter LTL in the general population. Maternal stress during pregnancy has also been associated with shorter telomeres in their offspring [25]. Concerning stress-related hormonal assessment in correlation to LTL, only cortisol reactivity to an acute psychosocial stress has been linked to shorter LTL in healthy children [26]. Psychological stress is increased in children [27] and adolescents with T1D [28], ranging from 17 to 50% of pediatric cases, and is accompanied by psychosomatic disturbances, disrupted behavior, and lower quality of life [28]. Persistent activation of the stress system can lead to further insulin resistance and aggravate glycemic control [29]. Parental or caregiver stress also seems to increase, while upon T1D diagnosis, most caregivers develop distress or experience depression [30]. This, in turn, afflicts family functioning [31] and diabetes outcomes [29]. Family and peer support are identified as important predictors of self-efficacy in managing the disease [32], alleviating stress, and providing children with inclusiveness, protection, and opportunities for socialization without discrimination.

Assessing LTL in a T1D pediatric cohort in comparison to age- and sex-matched healthy counterparts will corroborate the knowledge of telomere shortening in young patients with an autoimmune disease characterized by increased systemic inflammation and emotional stress and help explore any correlations of pediatric perceived stress and well-being, as well as hormonal and inflammatory markers, with LTL.

## 2. Results

### 2.1. Sociodemographic, Clinical and Other Descriptive Characteristics of Children and Caregiver Parents Who Participated in the Study

Age-matching between the two groups was carried out before group comparisons. No statistically significant difference was found concerning sociodemographic and descriptive characteristics between the two groups, the T1D group (n = 35) and the healthy control group (n = 35). Children with T1D had a mean age of 10.10 ± 2.10 years and were 60% females, while children in the control group had a mean age of 10.28 ± 1.81 years and were 54.3% females. The only baseline difference between the two groups was the HbA1c, as expected (7.40% ± 1.50 versus 5.40% ± 0.30, *p* < 0.001). Detailed descriptive participants’ characteristics are presented in Table 1 for each group. Parental/caregiver educational level did not differ between the two groups, but occupational status did differ (*p* = 0.029), with T1D parents/caregivers showing a greater percentage of unemployment (37.1%) versus control group parents/caregivers (14.3%). Furthermore, families of the control group were mainly recruited from Athens (77.1%) rather than the province, unlike T1D families (54.3% from Athens) (*p* = 0.044). Parental and demographic characteristics are shown in Table 2.

### 2.2. Relative LTL Differed Between Children with and Without T1D

Relative LTL was calculated as the T/S ratio. PCR analysis (mean of triplets with less than 10% of CV between triplicates) gave a value of 0.824 ± 0.134 for the T1D group and 0.894 ± 0.110 for the control group, showing that children with T1D had lower T/S ratio (*p* = 0.020) (Table 3), with a mean difference = 0.070 and Cohen’s d = 0.57, an effect size which is biologically meaningful. The T/S ratio did not differ among the 70 study children based on pubertal stage (*p* = 0.586), parental/caregiver educational level (*p* = 0.740), place of residence (*p* = 0.541), or level of physical activity (*p* = 0.916). It only differed according to biological gender, where males presented a statistically significantly higher T/S ratio (0.899 ± 0.126) than females (0.820 ± 0.121) (*p* = 0.022).

### 2.3. Relative LTL of Children with T1D Differed Based on Their Biological Gender

When groups were analyzed separately, the Independent *t*-test showed a significant within-group gender-related difference in the T1D group, with mean ± SD T/S ratio in males being higher (0.880 ± 0.131) versus females (0.787 ± 0.126, *p* = 0.042) (Table 4). Moreover, T1D females presented a lower T/S ratio contrary to healthy females (0.876 ± 0.098, *p* = 0.017), whereas between groups, males’ LTL showed no difference. No significant differences were, also, found concerning further biomarkers based on the biological gender (Table 4) in the T1D group. Within the T1D group, no other difference was found in the T/S ratio based on the presence of diabetic ketoacidosis at T1D onset (*p* = 0.442), use of CGM (*p* = 0.609), or occurrence of a severe hypoglycemic episode during the last six months (*p* = 0.820) (data not presented).

### 2.4. Laboratory Markers Differed Between Groups but Were Unrelated to Relative LTL

HbA1c, as expected, was higher in the T1D group with a value of 7.40 ± 1.50% versus 5.40 ± 0.30% in the control group (*p* < 0.001) (Table 1), and hsCRP was significantly higher in the T1D group (0.44 ± 0.79 mg/L) compared to the control group (0.19 ± 0.63 mg/L) (*p* = 0.030) (Table 3). The two groups did not significantly differ in other laboratory markers (i.e., serum cortisol and full blood count). In the T1D group, HbA1c and HsCRP were not correlated (r = 0.243, *p* = 0.160). HsCRP was found to have a significant positive correlation with BMI z-score (r = 0.407, *p* = 0.015) and a negative correlation with diabetes duration (r = −0.339, *p* = 0.047). Diabetes duration was, also, negatively correlated with serum cortisol (r = −0.355, *p* = 0.046) and WBC (r = −0.515, *p* = 0.002). Important intercorrelations are depicted in Table 5.

### 2.5. Pediatric and Parental Perceived Stress Did Not Differ Between Groups with and Without T1D

Parental/caregiver perceived stress (PSS) did not differ between the study groups (*p* = 0.505). This finding served to exclude parental general stress levels from confounding variables in further analysis. Parental perceived stress did not differ within groups according to pediatric pubertal stage or sociodemographic variables (residency, occupation, marital status, and educational level). It showed no correlation with any clinical milestones that might transiently elevate stress levels, such as children’s age, age of diagnosis, shorter diabetes duration, total WBC count, serum cortisol, or any other variable (Supplementary Material), but only showed a negative correlation with the child’s quality of life for all participants (PedsQL, total score) (r = −0.327, *p* = 0.006).

The children’s perceived stress (SiC, total score) and stress subcategories (distress, perceived social support, wellbeing) presented no significant difference between the T1D and control group. The child’s quality of life (PedsQL, total score) was significantly lower in the T1D (66.29 ± 9.62) versus the control group (73.34 ± 11.28) (*p* = 0.010) (Table 3). Moreover, in children with T1D, PedsQL was lower if the child’s distress (SiC) was higher (r = −0.338, *p* = 0.047) (Supplementary Material).

### 2.6. Analysis of Independent Factors That Might Interelate with the Lower rLTL in T1D Group

Correlation analysis was separately performed in the T1D group. The T/S ratio was positively correlated with the perceived presence of social support (*p* = 0.041) and marginally correlated with total perceived stress by SiC (*p* = 0.051), but it was not correlated with any other psychosocial or clinical measurements (Supplementary Material). Multivariate regression analysis with multiple model approaches was then performed to adjust for relevant covariates (age, gender, hsCRP, HbA1c, diabetes duration, BMI z-score, sociodemographic characteristics, parental age, psychosocial results). Intercorrelated variables were not entered in the same models (i.e., hsCRP and BMI z-score). The dominant model is presented in Table 5. The T/S ratio significantly decreases for female patients (beta = −0.493, *p* < 0.001), when the interaction term “gender X Social support” decreases (beta = 0.128, *p* < 0.002), and when diabetes duration increases (beta = 0.024, *p* = 0.045) after adjustment for the child’s age, parental age (meaning maternal age in 71.4%), parental occupational status, marital status, and educational level of caregiver parent. This model explained 49,7% of relative LTL in the T1D group (R-square = 0.497, *p* = 0.014, F = 3.094) (Table 5). No relevant variable was a significant predictor of the T/S ratio for the healthy control group (Supplementary Material).

### 2.7. Intercorrelation of Female Gender with Perceived Social Support Presents Increased rLTL in the T1D Group

**Figure 1:**
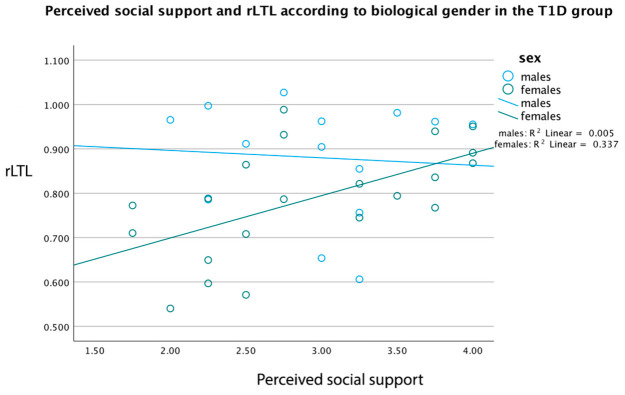
Correlation of relative LTL with perceived social support in children and adolescents with T1D, according to child’s biological gender. rLTL = relative LTL.

In the T1D group, females (n = 21) showed a significant positive correlation between LTL and social support (r = 0.581, *p* = 0.006), while in boys (n = 14), this relation was non-significant (r = −0.209, *p* = 0.474). As presented in Figure 1, females had a significantly increasing fit line of LTL X social support (y = 0.51 + 0.1 × x, *p* = 0.005), and males presented a slightly decreasing fit line of LTL X social support (y = 0.93 − 0.02 × x, *p* = 0.337). Multiple stratified analyses were performed to further investigate any rLTL differences based on the biological gender, such as the quality of life, residence, marital status, BMI z-score and hsCRP, but no significant differences emerged.

In Figure 1, female children (n = 21) present a significant positive correlation between relative LTL and perceived social support (r = 0.581, *p* = 0.006), whereas male children (n = 14) present a non-significant negative correlation between these two variables (r = −0.209, *p* = 0.474). 

## 3. Discussion

In the present study, relative leukocyte telomere length (rLTL) was significantly shorter in children and adolescents with T1D in comparison to age- and sex-matched healthy peers. This specific cohort of pediatric patients presented a gender-dependent reduction in rLTL, as females with T1D presented the lowest LTL compared to any other subgroup. It cannot be assumed that T1D differentially affects young female patients, but it cannot be excluded either. RLTL did not correlate with micro-inflammation nor with glycemic control but weakly correlated with diabetes duration and perceived social support, as a female-related feature. Lower LTL has been proven to be an independent predisposing factor for future coronary heart disease in children in general [33] and in patients with T1D in particular [21,34]. Therefore, in children and adolescents with T1D, it is of paramount importance to unravel factors that may prevent the shortening of telomeres in peripheral blood. Current results suggest an early emergence of telomere shortening in T1D patients, with apparently considerable intensity in females. To the best of our knowledge, this is the first study comparing rLTL between children/adolescents with T1D and age-matched healthy peers, taking psychological stress into account. Since heritability is a major determinant of LTL, it was partly controlled with the inclusion of siblings in each group (by 68.6%).

Shorter telomeres have been previously found in adults with T1D [13,16] and in children with impaired glucose metabolism [35,36]. Causality between T1D and shorter LTL cannot be proven, but reverse causality cannot be excluded since altered TL can change gene expression [37,38], and thus immune or endocrine functions. Indeed, the risk of carrying shorter TL has been found to be a predisposing risk factor for T1D through Mendelian randomization analysis using data obtained from the open GWAS database [19]. In the TEDDY study, children of high-prevalence countries for T1D, such as Finland and Sweden, presented shorter TL [22], implying that shorter TL may precede T1D emergence. On the other hand, the recurrent immunological activation, in the course of auto-immune disease, could alter T-lymphocytes’ dynamics and result in inflammation-related senescence [34]. Furthermore, how telomeres change over time also seems to depend on intrinsic (birth) LTL, where longer LTL is followed by greater attrition in the long run [20,22,39,40]. Moreover, telomere stability or elongation over time has been reported as a paradox and could be explained by telomerase-mediated actions [34,41] or the upregulation of telomerase activity, antioxidant cell capacity [19,21], and multiple other compensatory mechanisms, such as the improvement of the DNA damage repair system [19]. Although the pace of LTL attrition in relation to age in children is less pronounced [18,35], all the above suggest that rLTL in T1D patients measured at a single timepoint should be interpreted with caution, inspected as predating to T1D or as an epigenetic feature, under a stressful or oxidative environment.

On secondary analysis, the appearance of shorter telomeres in our T1D children was correlated only with gender and social environment (perceived support), but not with HbA1c, TIR, glucose variability, hsCRP, diabetes duration (only weakly), DKA at diagnosis, BMI z-score, or age. These findings point towards the role of other mediating T1D-related pathways on LTL shortening, probably related to the female gender. Concerning chronic hyperglycemia, the longitudinal pediatric study of Tesovnic and colleagues showed an accelerated telomere attrition according to normalized diabetes duration with age for those Slovenian children with T1D and persistently poor glycemic control (mean HbA1c 9.83% ± 0.94) compared to those with good glycemic control (mean 6.92% ± 0.45) after seven years of monitoring [18]. Gender difference is not reported. Cross-sectional adult studies [13,16] have shown that increased HbA1c or inflammation was not related to LTL of T1D patients, in accordance with our results. Here, HbA1c did not differ between the two genders. A highly oxidative cell environment, caused by chronic hyperglycemia, is expected to induce a direct telomere affliction, because telomeric repeats are rich in guanine residues and exceptionally prone to oxidation by ROS [42]. The above explains a cumulative effect of longer diabetes duration on LTL. Hyperglycemia is proven a non-disputable, although not exclusive, risk factor for telomere shortening in T1D patients, and we suggest that the median HbA1c of 7.50% ± 1.50 in our cohort, delineating satisfactory glycemic control, could partly moderate any rLTL attrition.

The weak positive correlation of diabetes duration with LTL was in the opposite direction compared to some studies [16], but not all [13,20], concerning long-term survivors. In the longitudinal pediatric study by Tesovnic et al., a correlation of T1D duration with LTL is not reported per se, but only the normalized diabetes duration with age differed according to glycemia. Thus, the literature remains ambiguous in how T1D duration is connected to LTL, but longitudinal data are needed to explain the long-term effect of T1D duration on LTL. From this perspective, the apparently longer LTL with longer T1D duration in our pediatric T1D group could be rationally attributed to an assumable survivor bias based on the relatively short diabetes duration (2.00 ± 2.49 years), frequently observed at this stage of the disease progress, where the partial remission phase can moderate insulin needs. Νitrosative burden and increased glucose variability can affect LTL dynamics from T1D onset [18], but insulin therapy and long-term adjustment in daily doses serve to stabilize LTL or affect other compensation mechanisms, as proposed by Guanping Wei and colleagues [19], and therefore counterbalance initial LTL shortening. In our cohort, patients with shorter T1D duration also presented higher cortisol levels, immune activation, and inflammation. This clinical profile can be explained by the “metabolic telomere attrition hypothesis”, according to which telomere attrition constitutes an energy trade-off in metabolically expensive periods, driven by salvage biological functions [43]. The cross-sectional analysis of our data and the variability of the oxidation status [14] should be considered. Other laboratory markers, such as 8-hydroxy-29 deoxyguanosine, 8-nitroguanine, sE-selectin, or AGEs, could be more indicative in the T1D research [15,16].

A gender-specific difference observed in our T1D children’s rLTL was in the opposite direction according to the literature, as female children of the general population [8,36] and females of reproductive age [20,44] display longer TL. Estrogens control telomerase activity through direct and indirect actions on human telomerase reverse transcriptase (hTERT) and promote lengthening or counterbalance shortening of telomeres [9,45]. In newborns, no gender difference has been previously found [46]. However, a meta-analysis published in 2016 reported that female participants with diabetes presented shorter TL compared to male participants [14] in accordance with our findings; thus, females with T1D may carry an LTL vulnerability. Recently, Wong and co-workers presented that although carrying longer LTL compared to male children, females’ beta-cell function was negatively associated with LTL [36]. Counter-regulatory mechanisms could be hypothesized to be implicated in female children’s telomere dynamics. Sexual dimorphic susceptibility to metabolic disorders, driven by the intrauterine environment, is well documented in the literature [47], but in T1D, a further elucidation of the perinatal environment is required. It is important to highlight that T1D subjects with greater LTL at baseline display greater attrition in the long run [20].

The gender-related difference in rLTL of our cohort could not be attributed to any other difference in biological markers. Higher rLTL was intercorrelated with higher perceived social support (subscale of the perceived stress assessment) and remained significant only as a gender-related feature after controlling for key variables (children’s age and parental age) and further sociodemographic factors. Whether social support is protective primarily for female T1D patients rather than males should be interpreted with caution due to the small subgroup size. Social support stands for “psychological and material resources” contributing to one’s ability to deal with stress and offering a supportive environment for emotional expression [48]. In this study, it was assessed through questions about emotional externalization, feelings of social belonging, and main aspects of coping [49]. Previous studies have pointed out a possible impact of social support on LTL, as shorter telomeres have been consistently connected to lower social support in children of the general population [50], to internalizing attitudes expressed by preschool children [25], and to lower feelings of attachment at school in adolescents [51], with no gender diversity been reported. Drury and co-authors have reported that exclusively girls present shorter LTL along with increased exposure to intrafamily violence (witnessing) or other adverse events, while in boys, such an association could not be confirmed [52], a finding which suggests a different biological processing in stressful events. On the contrary, TL of saliva samples assessed in nine-year-old boys showed a significant decline when they experienced an adverse family environment or multiple transitions in family structure [53], implying that boys can be biologically sensitive to psychosocial adversity.

From a socio-cultural aspect, research indicates that young women are more vulnerable to a lack of social support and prioritize strong relationships to achieve better mental health [54,55]. From this perspective, female youths could be regarded as a vulnerable population, because they present greater diabetes-specific stress levels versus males [27], which was not assessed here and therefore cannot be excluded as an underlying factor. Diabetes-specific and general stress affect primary diabetes outcomes (quality of life, glucose management) significantly but in different ways [28]. A meta-analysis of 121,432 children and adolescents evaluating the effect size of social support on their well-being showed a significantly stronger effect size in female participants versus males [56], pointing out the importance of social support for females. An interesting finding was described by Gecková and colleagues, where European adolescent females reported higher social support than males, together with more unfavorable psychological and psychosomatic health outcomes [57], showing gender-specific patterns of stress coping and emotional expression. From a biological perspective, sexual dimorphism is depicted in cortisol stress reactivity and cortisol metabolism already in childhood, but these patterns remain inconclusive [58]. Furthermore, sex-specific vulnerability is presented in the pathophysiology of common endocrinopathies, which is regarded to be directed by genes, hormones, and the environment [59]. Since most studies conducted in children assess poverty, bullying victimization, childhood maltreatment, or exposure to intra-family violence [52,60], which are regarded as harsh versions of chronic stress, future large-scale studies assessing LTL in pediatric T1D could incorporate the assessment of disease-specific stress and perceived social support through specialized questionnaires. Due to the small sub-group sizes in our study, any gender-specific finding should be regarded as hypothesis-generating, rather than definitive.

### Strengths and Limitations

Important strengths of this study are the focus on a rare pediatric group, which is understudied concerning LTL; the recruitment of an age-matched healthy control group, which also included siblings to partly adjust for heritability and other confounding factors; and the robust telomere assay method with the incorporation of an internal DNA sample control. MM-qPCR is a commonly used method, with a vast agreement to be used, at least, in adults [9] because it is a relatively easy, fast, and cheap procedure; does not require much DNA quantity; and can produce high-throughput results [61]. In our sample, cases and controls did not differ in terms of the absolute number of WBC (Table 3), but individual leukocyte distribution/composition of the whole blood sample [6,62] or fluctuations were not determined here, which can dynamically affect human telomere biology [40,41]. Our EDTA blood samples were tested for integrity, but the blood sampling method, storage, and possible DNA degradation may have affected DNA quality or accounted for qPCR discrepancies [62]. Moreover, there are considerations about the comparable qPCR performance, validity, and precision versus the reference method (Southern blot), when applied to patient populations [63]. Finally, Gardner et al. have stated that the PCR assay method could be less suitable for tracing subtle LTL differences concerning sex or age [44].

The small study sample raises concerns about the statistical power of any association and regression analysis, as well as about any generalization of our findings. One-point measurement of HbA1c is not representative for the assessment of long-term T1D glycemic control; thus, large-scale multiple-time measurements in longitudinal studies should be implemented. Only general stress levels were assessed in our protocol to enable comparison with healthy controls. Disease-specific pediatric and parental stress could possibly yield further secondary outcomes [27]. Divergent stress types (i.e., trauma, mild daily distress, social deprivation, familial instability, discrimination) seem to differentially affect LTL attrition, depending on their chronicity, acuity, recurrence, strength, and timing [24]. Moreover, we did not evaluate the sociodemographic characteristics separately for both parents [23] nor paternal age [22]. Recruitment of siblings in the control group carries the negative impact of the psychological and behavioral adjustments of all family members after T1D diagnosis in one family member [64] and may impact families’ psychometrics. Finally, children’s diet and eating behaviors were not studied. Mediterranean diet and the intake of foods rich in antioxidants and phytochemicals, or a reduction in body weight, could reverse LTL status [11,65].

## 4. Materials and Methods

### 4.1. Study Design

This is a cross-sectional clinical study of children and adolescents with T1D vs. age-matched healthy controls. The primary objectives were the assessment of leukocyte telomere length (LTL) in participants with T1D in comparison to age-matched healthy controls. Secondary objectives were the correlation of children’s LTL with levels of pediatric perceived stress, glycated hemoglobin (HbA1c), morning serum cortisol, and high-sensitivity CRP (hsCRP). The study protocol was approved by the Scientific and Ethics Committee of the “Aghia Sophia” Children’s Hospital of Athens, and the Institutional Review Board of the Medical School of Athens, Greece, with an approval number 45274 on 31 August 2020. All participants were included in the study only after obtaining written consent from their parents and after being individually and fully informed about the purposes and procedures of the research. Patients’ and parents’ personal data collected were kept confidential and available only to the researchers.

### 4.2. Participants

The study population consisted of a total of 70 children (35 participants with T1D and 35 age-matched healthy controls) aged 6–13 years and one caregiver parent per child. Children with T1D were followed at the Diabetes Center of the Division of Endocrinology, Diabetes and Metabolism of the First Department of Pediatrics of the National and Kapodistrian University of Athens (NKUA) Medical School, and age-matched healthy controls were either siblings of children with T1D or children followed at the same Division for growth evaluation, at the “Aghia Sophia” Children’s Hospital of Athens (Greece), from April 2021 to May 2022. Participants were recruited during their regular clinical visit or via telephone contact by the researcher. Only one parent of the family participated in the study. Each parent accounted for 1 or 2 children (one in the T1D group and the other in the control group). Pediatric patients with T1D were all on insulin regimen. Inclusion criteria for the T1D group were as follows: (a) diagnosis at least 6 months before inclusion in the study, (b) absence of T1D-related complications, (c) absence of other endocrine disorders (i.e., thyroiditis) or temporary illness, (d) residents of Greece, and (e) good communication skills in the Greek language. Inclusion criteria for the control group were as follows: (a) absence of any chronic disease, (b) residents of Greece, and (c) good communication skills in the Greek language. In order to eliminate the possibility of hsCRP elevation as a result of intercurrent illness, children and adolescents with recent infections (i.e., COVID-19) or vaccination were excluded. Primer calculation for the study sample was based on the formula n = (Z^2^ × σ^2^)/Ε^2^, n sample size, Z value for confidence level (1.96), σ = estimated variability (SD) (we regarded estimated variability in T1D children population for T/S ratio SD = 0.15), E = margin of error, 5%.

### 4.3. Biomarker Collection

In the morning (8.00–8.30 am), peripheral blood was collected and stored in tubes with EDTA at 4 °C and then at −80 °C until analysis. Leukocyte telomere length (LTL), morning serum cortisol, hsCRP, HbA1c, and other routine biochemical markers were assessed for each child/adolescent. Tubes without EDTA were sent immediately for centrifuging, and serum was collected and placed for long-term storage at −20 °C. Serum hsCRP was assessed via the immunoturbidity assay method, using a Cobas 6000 (c501) analyzer (GmbH, Mannheim, Germany), with a detection limit of 0.15 mg/L. The kit was CRP LX HS (Roche Diagnostics GmbH, Mannheim, Germany), 300Tests, Cobas c, Integra. Intra-assay CV was <4% and inter-assay CV was <6.8%. Serum cortisol was measured based on the electrochemiluminescence immunoassay principle at a Cobas e411 analyzer Roche Diagnostics (GmbH, Mannheim, Germany), with an analytical sensitivity of 0.054 µg/dL. All biochemical assays were conducted blinded to other participant characteristics.

### 4.4. DNA Isolation

Standardized protocols for DNA extraction, storage, and handling were followed to preserve the integrity of the samples and minimize telomere degradation. Genomic DNA was extracted from fresh peripheral whole blood using the NucleoSpin^®^ Blood Kit (Macherey-Nagel, GmbH & Co. KG, Düren, Germany) according to the manufacturer’s instructions. Blood samples were collected only for this study’s purposes and remained for one year or less at 4 °C. All extractions and downstream handling were performed on ice or at 4 °C to minimize enzymatic activity and reduce DNA degradation. DNA samples were subjected to agarose gel electrophoresis to assess their integrity and to confirm the absence of fragmentation. High-concentration stock solutions of DNA samples were stored at −80 °C for long-term storage. The concentration of purified leukocyte DNA was spectrophotometrically determined. Diluted samples were prepared prior to qPCR analysis. Vortexing and pipetting were minimized to reduce mechanical shearing of telomeric regions.

### 4.5. qPCR Measurement

Relative telomere length was determined by mm-qPCR based on a previously published protocol by Cawthon [66], using a real-time PCR Foundation LightCycler 96 analyzer, Roche Diagnostics, Mannheim, Germany [67] at the Genetics Laboratory of BRFAA, Greece. A ratio of telomere-repeat-copy number to single-copy gene number (T/S ratio) was determined and compared with a reference DNA (control) sample. Analyses were performed in triplicate, using a telomere-specific amplicon primer set and a single-copy gene amplicon primer set. For the relative quantification of leukocyte telomere length (LTL), albumin was selected as the reference gene. This allows for distinct and independent detection of the fluorescent signal from each amplicon during the melt curve analysis.

The samples were processed in batches. It was not feasible to analyze all samples within a single PCR run. Each 96-well plate included triplicate reactions per sample, plus the standard reference sample used to generate a standard curve for relative quantification. To mitigate potential batch effects, each PCR plate was loaded with an equal number of case and control samples. Case-control pairs were systematically distributed across all runs to minimize any potential bias due to batch processing. The intra- and inter-assay coefficients of variation were calculated as the standard deviation divided by the mean value of the replicate measurements. The intra-assay CV across all samples was ≤1.966%, indicating excellent reproducibility within runs. For the inter-assay variation, a subset of 7 patient samples was reanalyzed on a different day and in separate positions within the PCR plate. The inter-assay CV in this subset was calculated to be <5.96%.

Threshold cycle values were calculated from the raw data produced, using an external qPCR package, QuantStudio^TM^ Design & Analysis Software v1.5.3, that performs sigmoidal model selection from real-time qPCR data analysis to produce Ct values. Quality control parameters used for the amplifications include a cut-off of 0.10 for the replicative SD (standard deviation) of comparative cycle threshold triplets. Samples of purified DNA of the HeLa cell line, and SD1, SD2 (reference DNA obtained from a PCR-based telomere measurement kit of ScienCell Research Laboratories, Carlsbad, CA, USA) were added as internal controls in each PCR run of the 96-Well Reaction Plate (0.1 mL).

### 4.6. Clinical Examination and Demographics

Pubertal status was evaluated according to the Tanner staging during a complete clinical examination for all participants by a pediatric endocrinologist. Anthropometric measurements were also carried out (body weight, height, BMI, blood pressure). Children’s BMI was standardized by conversion to z-score (BMI z-score) as defined by age and gender, using the Centers for Disease Control and Prevention (CDC) growth charts [68]. Sociodemographic data were collected from the caregiver’s parent (gender, age, race/nationality, marital status, place of residence, educational level, and occupational status of caregiver), and children’s lifestyle data (physical activity level) were reported during the interview with the caregiver parent. Data about incidence of diabetic ketoacidosis at diagnosis and severe hypoglycemic episodes during the last 6 months were retrieved from pediatric medical records.

### 4.7. Questionnaires

Children’s perceived stress was assessed through the self-report “Stress in Children” (SiC) questionnaire [49,69]. It is a 21-question instrument designed for school-age children, aiming to assess perceived stress. Three components were evaluated: “degree of perceived distress (distress)”, “presence of well-being”, and “presence of social support”. Health-related quality of life in children was measured with the validated children’s version [70] of the Pediatric Quality of Life Inventory™ (PedsQLT™) 4.0 Generic Core Scales [71]. The diabetes version of this tool (PedsQL™ 3.0 Diabetes Module) [72] has also been validated in a Greek pediatric population with T1D [73], showing a lower diabetes-related quality of life for these children compared to their healthy peers. Children under the age of 8 years old completed the questionnaires with a guidance. Assessment of parental stress levels was evaluated by a general stress instrument, the Perceived Stress Scale (PSS 14) [74], validated in Greek with sufficient psychometric capacity [75]. It was selected in order to enable comparison between the two study groups and in order to assess and exclude those caregivers under excessive family stress conditions from our cohort.

### 4.8. Statistical Analysis

The study sample was age-matched with a tolerated age difference of ±1 year between the two groups (T1D and healthy controls). Participants’ data were presented as percentages for categorical variables and as mean ± standard deviation, or median ± interquartile range for quantitative variables, if not normally distributed. Kolmogorov–Smirnov or Shapiro–Wilk tests were used to check normality. The existence of a linear correlation between variables was assessed with Pearson or Spearman rho correlation coefficients. Necessary parametric or non-parametric tests (Independent Samples *t*-test and Mann–Whitney U) or the Chi square test were used, while ANOVA or Kruskal–Wallis analysis of variance was applied for variables with more than two categories. Due to the small study sample, variables carrying more than 2 categories were grouped into bivariate categories. Linear regression analysis was executed to investigate any associations between LTL and key psychosocial and clinical variables concerning children and caregivers, separately for the T1D group. Particularly, BMI z-score, pubertal stage, physical activity levels, diabetic ketoacidosis at diagnosis, recent severe hypoglycemic episode (last 6 months), parental age, marital status, and employment status of the caregiver parent were considered as variables. All of the consecutive regression models were adjusted for age and biological gender of children/adolescents to account for their confounding effect and improve model accuracy. For all analyses, *p* < 0.05 was considered statistically significant.

## 5. Conclusions

The current study can be regarded as a pilot study that confirms the early emergence of lower LTL in pediatric patients with T1D, in a sexual dimorphic pattern, unrelated to age, BMI, glycemic control, or inflammation status, and pointing towards diverse confounding factors, such as perceived social support. These findings should be treated as hypothesis-generating for future research. Longitudinal studies conducted in larger T1D pediatric cohorts, incorporating genetic variability, are needed to clarify the role of gender, psychosocial support, diabetes duration, and long-term glycemic control on telomere length dynamics.

## Figures and Tables

**Table 1:** Sociodemographic and other descriptive characteristics for each group, T1D, and age-matched healthy controls.

Characteristics	T1D Group (n = 35)	Control Group (n = 35)	*p* Value (Between Groups)
Sociodemographic and clinical			
Age (years), mean (SD)	10.1 (2.1)	10.3 (1.8)	0.693
Child’s gender, n (%)			
Female	21 (60.0)	19 (54.3)	0.629
Male	14 (40.0)	16 (45.7)	
Family History of T1D, n (%)			
First degree relative (siblings)	-	24 (68.6)	-
General population	-	11 (31.4)	
Pubertal status, n (%)			
Prepubertal	14 (40.0)	19 (54.3)	0.147
Pubertal	21 (60.0)	14 (40.0)	
Body weight (kg), mean (SD)	40.62 (14.61)	40.14 (15.22)	0.895
Height (m), mean (SD)	1.42 (0.15)	1.43 (0.15)	0.694
BMI (kg/m^2^), median (IQR)	18.43 (6.23)	17.43 (4.28)	0.271
BMI z-score, median (IQR)	−0.19 (1.42)	−0.42 (0.98)	0.541
Systolic Blood Pressure (mmHg), mean (SD)	107.1 (10.7)	110.4 (11.3)	0.225
Diastolic Blood Pressure (mmHg), mean (SD)	66.1 (9.6)	69.5 (12.3)	0.208
Heart Rate, mean (SD)	86.5 (14.6)	85.9 (10.6)	0.836
HbA1c, median (IQR)	7.40 (1.5)	5.40 (0.3)	**<0.001**
Physical Activity Level, n (%)			
Never/Rarely (0–1 times/week)	15 (42.9)	11 (31.4)	0.458
Frequently/very frequently (≥2 times/week)	20 (57.1)	24 (68.6)	
Special characteristics of T1D children (n = 35)			
Age of T1D onset, mean (SD)	7.45 (2.59)	-	-
Diabetes duration in years, median (IQR)	2.00 (2.49)	-	-
Diabetic ketoacidosis at diagnosis, n (%)			
No	14 (43.7)	-	-
Yes	18 (56.3)		
Method of glucose self-measuring, n (%)			
Self-Monitoring Blood Glucose (SΜBG) (%)	7 (20.0)	-	-
Continuous Glucose Monitoring System (CGMS) (%)	25 (71.4)		
Sensor-augmented pump (SAP)	3 (8.6)		
Glucose monitoring of the last 15 days, for children on CGMS (n = 28)			
Time in Range (TIR), mean (SD)	63.70 (18.60)	-	-
Time above Range (TAR), mean (SD)	33.41 (18.92)	-	-
Time below Range (TBR), median (IQR)	2.00 (2.00)	-	-
Coefficient of Variance (CV), mean (SD)	35.19 (6.00)	-	-

Standard deviation: SD, IQR: interquartile range, n: number of individuals, T1D: type 1 diabetes, kg: kilograms, BMI: body mass index, SMBG: Self Blood Glucose Monitoring, CGMS: Continuous Glucose Monitoring System, SAP: Sensor Augmented Pump, TIR: time in range, TBR: time below range, TAR: time above range, CV: coefficient of variance. Chi square (Χ^2^), Independent Sample *t*-test, Mann–Whitney. Level of statistical significance at *p* < 0.05. Bold stands for statistical significance.

**Table 2:** Sociodemographic and descriptive characteristics of parents/ caregivers for each group, T1D, and age-matched healthy controls.

Characteristics	T1D Group (n = 35)	Control Group (n = 35)	*p* Value (Between Groups)
Age of caregiver, median (IQR)	43.0 (8.0)	42.0 (5.0)	0.850
Age category of caregiver, n (%)			
26–45	23 (65.7)	28 (80.0)	0.282
46–65	12 (34.3)	7 (20.0)	
Sex of caregiver, n (%)			
Female	25 (71.4)	29 (82.9)	0.255
Male	10 (28.6)	6 (17.1)	
Marital Status, n (%)			
Married	29 (82.9)	33 (94.3)	0.260
Divorced, Unmarried, Widow	6 (17.1)	2 (5.7)	
Place of Residence, n (%)			
Attica	19 (54.3)	27 (77.1)	**0.044**
Province	16 (45.7)	8 (22.9)	
Educational Status, n (%)			
Up to secondary	22 (62.9)	18 (51.4)	0.469
Higher than secondary	13 (37.1)	17 (48.6)	
Employment status, n (%)			
Employee, civil servant, freelancer	22 (62.9)	30 (85.7)	**0.0** **2** **9**
Unemployed, Retired	13 (37.1)	5 (14.3)	

IQR: interquartile range, n: number of individuals, T1D: type 1 diabetes. Chi square (Χ^2^), Mann–Whitney. Level of statistical significance at *p* < 0.05. Bold stands for statistical significance.

**Table 3:** Telomere to single-copy gene (T/S) ratio, laboratory markers, perceived childhood stress, parental perceived stress, and quality of life in children with T1D (n = 35) and age-matched healthy controls (n = 35).

Characteristics	T1D Group (n = 35)	Control Group (n = 35)	*p* Value (Between Groups)	Lower 95% CI	Upper 95% CI	Cohen’s d
Laboratory values						
T/S ratio, mean (SD)	0.824 (0.134)	0.894 (0.110)	**0.020**	−0.128	−0.011	−0.57
Cortisol, (morning, serum), median (IQR)	10.55 (5.96)	9.73 (6.20)	0.559	−1.85	3.42	0.15
High Sensitivity CRP (mg/L), median (IQR)	0.44 (0.79)	0.19 (0.63)	**0.031**	−0.19	1.71	0.38
WBC × 1000/μL, mean (SD)	6.10 (1.25)	6.52 (1.53)	0.218	−1.08	0.25	−0.30
RBC × 1,000,000/μL, median (IQR)	4.89 (0.53)	4.87 (0.43)	0.444	−0.63	0.31	−0.16
Hct%, mean (SD)	39.80 (2.25)	38.89 (2.27)	0.113	−341.78	−256.55	−3.35
Estimated Perceived Stress, Children and Parents						
SiC, Total score, mean (SD)	2.67 (0.37)	2.61 (0.27)	0.441	−0.10	0.22	0.19
SiC, Presence of wellbeing, mean (SD)	2.70 (0.34)	2.72 (0.50)	0.574	−0.15	0.26	0.14
SiC, Presence of social support, mean (SD)	2.94 (0.69)	2.92 (0.51)	0.883	−0.27	0.31	0.04
SiC, Distress, median (IQR)	2.22 (0.44)	2.22 (0.44)	0.995	−0.10	0.16	0.12
Perceived Stress Scale (PSS), median (IQR)	22.94 (8.05)	21.49 (7.13)	0.505	−2.17	5.09	0.19
Estimated Quality of Life, Children						
PedsQL Total score, mean (SD)	66.29 (9.62)	73.34 (11.28)	**0.010**	−12.32	−1.77	−0.64
PedsQL Diabetes Module						
PedsQL-Diabetes, mean (SD)	58.70 (15.07)	-				
PedsQL-Treatment Barriers, median (IQR)	75.00 (25.00)	-				
PedsQL-Treatment Adherence, mean (SD)	66.53 (14.34)	-				
PedsQL-Worries, median (IQR)	66.67 (33.33)	-				
PedsQL-Communication, median (IQR)	66.67 (33.33)	-				
PedsQL Healthy Module						
PedsQL-Health, mean (SD)	-	79.14 (15.07)				
PedsQL-Emotions, mean (SD)	-	63.82 (16.88)				
PedsQL-With others, mean (SD)	-	76.91 (14.72)				
PedsQL-School, mean (SD)	-	71.91 (12.91)				

T/S: telomere/single-copy g-ene ratio, standard deviation: SD, IQR: interquartile range, n: number of individuals, WBC: white blood cells, RBC: red blood cells, Hct: hematocrit, PSS: Perceived Stress Scale, SiC: Stress in Children, PedsQL: Pediatric Scale Quality of Life. Chi square (Χ^2^), Independent Sample *t*-test, Mann–Whitney U. Level of statistical significance at *p* < 0.05. Cohen’s d shows the effect size of the differences between means. Bold stands for statistical significance.

**Table 4:** Gender differences in relative LTL and other biological markers between and within the T1D and the healthy control group.

Biological Markers	Gender	T1D Group	Control Group	*p*-Value
**T/S ratio**	Females	0.787 (0.126)	0.876 (0.098)	**0.017**
	Males	0.880 (0.131)	0.915 (0.122)	0.547
	*p*-value	**0.042**	0.308	
**Serum cortisol**	Females	11.64 (4.21)	9.27 (6.02)	0.128
	Males	11.95 (5.98)	10.08 (9.77)	0.963
	*p*-value	0.969	0.270	
**HsCRP**	Females	1.19 (2.28)	0.26 (1.02)	0.363
	Males	1.93 (2.91)	0.17 (0.56)	0.051
	*p*-value	0.447	0.554	
**WBC**	Females	6.20 (1.40)	6.04 (2.03)	0.401
	Males	5.96 (1.04)	6.54 (2.79)	0.561
	*p*-value	0.585	0.855	
**BMI z-score**	Females	0.80 (1.11)	−0.02 (0.92)	0.473
	Males	0.52 (1.03)	−0.53 (0.99)	0.146
	*p*-value	0.941	**0.037**	
**HbA1c**	Females	7.29 (0.97)	5.40 (0.20)	**<0.001**
	Males	7.75 (1.28)	5.40 (0.53)	**<0.001**
	*p*-value	0.573	0.600	

T/S: telomere/single-copy gene ratio. Independent Sample *t*-test, Mann–Whitney. Level of significance at *p* < 0.05. Bold stands for statistical significance. Participants’ biological gender in the T1D group: n = 21 females and n = 14 males (total n = 35). Participants’ biological gender in the healthy control group: n = 19 females and n = 16 males (total n = 35).

**Table 5:** Regression analysis on parameters related to T1D children’s relative LTL (n = 35).

	Coefficients ^a^						
Independent Variables ^b^	Unst. Coeff		Stand Coeff	t	*p*-Value	Collinearity Statistics	
	B	SE	Beta			Tol	VIF
**Child’s gender**	−0.493	0.119	−1.809	−4.130	**<0.001**	0.105	9.544
**Child’s age**	−0.006	0.011	−0.099	−0.597	0.556	0.732	1.365
**Parental age**	0.003	0.004	0.115	0.707	0.486	0.763	1.311
**T1D duration**	0.024	0.011	0.327	2.112	**0.045**	0.838	1.193
**Gender X Support**	0.128	0.037	1.485	3.492	**0.002**	0.111	8.997
**Parent occupation**	0.013	0.045	0.049	0.299	0.768	0.758	1.319
**Parent education**	−0.027	0.050	−0.097	−0.547	0.589	0.640	1. 562
**Marital status**	0.123	0.061	0.348	2.025	0.054	0.679	1.474

^a^ Dependent variable: relative LTL (lymphocyte telomere length). ^b^ Covariates: biological gender, age, diabetes duration, ‘gender X social support’, parental age, parental occupation, parental education, marital status. Gender: 0 = female, 1 = male. Regression analysis in three consecutive models. R square = 0.497, *p* = 0.014, F = 3.094. Bold means statistical significance. Statistical significance level at *p* < 0.05.

## Data Availability

The original contributions presented in this study are included in the article/Supplementary Material. Further inquiries can be directed to the corresponding author.

## Supplementary Materials

The following supporting information can be downloaded at: https://www.mdpi.com/article/10.3390/ijms27093895/s1.

## Abbreviations

The following abbreviations are used in this manuscript:

T1D	Type 1 Diabetes
DM	Diabetes Mellitus
LTL	Lymphocyte Telomere Length
T/S	Telomere/single copy gene Ratio
BMI	Body Mass Index
HsCRP	High-Sensitivity C-Reactive Protein
AGEs	Advanced Glycated End Products
qPCR	Quantitative Polymerase Chain Reaction
MM-qPCR	Monochrome multiplex quantitative real-time PCR
Flow-FISH	Fluorescence in-situ Hybridization with Flow Cytometry
SiC	Stress in Children
PSS	Perceived Stress Scale
PedsQL	Pediatric Scale of Quality of Life
SBP	Systolic Blood Pressure
DBP	Diastolic Blood Pressure
ROS	Reactive Oxygen Species
WBC	White Blood Cells
