# Supplementary material for: Relative Leukocyte Telomere Length Is Shorter in Children and Adolescents with Type 1 Diabetes: Screening of Basic Psychosocial Aspects
Source: Int J Mol Sci. 2026 Apr 27;27(9):3895. doi: 10.3390/ijms27093895 (PMC13164480; doi:10.3390/ijms27093895)
Supplement: Supplementary file 1 [file ijms-27-03895-s001.zip › Supplementary material_Extra Information in methodology and results.pdf]

1. Extraction of DNA from peripheral blood samples

After preparation, the blood samples were lysed, according to the following procedure. Pipette 25μL of Proteinase K and up to 200μL of blood (equilibrated at room temperature) into 1.5mL microcentrifuge tubes. 200 μL of buffer B3 were added to the samples and the mixtures were shaken vigorously (10–20 s). The samples were then incubated at 70 °C for 30 minutes and vortexed several times during this step. To achieve DNA binding conditions 210 μL of ethanol (96–100 %) were added to each sample and vortexed again. To bind the DNA in each preparation, a NucleoSpin® blood column placed in a collection tube was taken and the sample loaded. This was followed by centrifugation for 1 min at 11,000 x g. The silica membrane was washed and then the NucleoSpin® blood column was placed in a new collection tube (2 mL) where 500 μL of Buffer BW were added. Centrifugation was performed for 1 min at 11,000 x g followed by a second washing of the silica membrane. The NucleoSpin® blood column was placed in a new collection tube (2 mL), 600 μL buffer B were added, followed by centrifugation for 1 min at 11,000 x g. Residual ethanol was removed during this step. To elute the pure DNA, the NucleoSpin® blood column was placed in a 1.5 mL microcentrifuge tube and 100 μL of pre-warmed BE buffer (70 °C) were added. The buffer solution was dispensed directly onto the silica membrane. This was followed by incubation at room temperature for 1 min and centrifugation for 1 min at 11,000 x g.

2. Telomere length measurement protocol

The biological blood samples were delivered to the respective laboratories of the "Aghia Sophia" Children's Hospital for biomarker analysis (biochemical laboratory), while a part of the blood samples with anticoagulant in windrops, were kept in refrigerator at 4°C for one year, so that it could be sent coded for telomere length analysis at the Genetics Laboratory at the Biomedical Research Foundation of Academy of Athens, where they were kept at -80° for long term storage. After DNA isolation, leukocyte telomere length (LTL) was measured by real-time PCR on the PCR Foundation Light Cycler 96 analyzer Roche Diagnostics, Mannheim, Germany, at the Genetics Laboratory of BRFAA, Athens. [1]. Analysis was performed using the Cawthon's Protocol [2], which is designed to directly measure the average telomere length of a population of human cells. A ratio of telomere-repeat-copy number to single-copy gene number (T/S ratio) was determined, and compared with a reference DNA (control) sample. Analyses were performed in triplicate, using a telomere-specific amplicon primer set and a single-copy gene amplicon primer set (albumin), as follows:

The primer sequences (5′ → 3′) for telomere amplification were:

telg (G rich) ACACTAAGGTTTGGGTTTGGGTTTGGGTTTGGGTTAGTGT

and
telc (C rich) TGTTAGGTATCCCTATCCCTATCCCTATCCCTATCCCTAACA.

and for amplification of the single copy reference gene albumin were:
albu (alb upstream) (CGGCGGCGGGCGGCGCGGGCTG GGCGGAAATGCTGCACAGAATCCT)
and
albd (alb downstr) (GCCCGGCCCGCCGCGCCCGT CCCGCCGGAAAAGCATGGTCGCCTGTT).
Each reaction consisted of 12μL of a 2× master mix (SYBR® FAST qPCR Kits, Kapa Biosystems), 1μM of each of the primers (telg, telc, albu, albd) and 20ng of purified genomic DNA to yield a final reaction volume of 20μL. Reagent and sample dispensing were carried out by a semi-automated liquid handling system (epMotion 5073, Eppendorf). Reactions were performed in 96-well plates, in four consecutive experiments, adjusted to group effect. Each plate contained a 6-point standard curve from 6.25ng to 100ng of genomic DNA. The thermal cycling profile was Stage 1: 15 min at 95°C; Stage 2: 2 cycles of 15 s at 94°C, 15 s at 49°C; and Stage 3: 32 cycles of 15 s at 94°C, 10 s at 62°C, 15 s at 74°C with signal acquisition, 10 s at 84°C, 15 s at 88°C with signal acquisition. The 74°C reads provided the C_t values for the amplification of the telomere template (in early cycles when the single copy gene signal is still at baseline); the 88°C reads provided the C_t values for the amplification of the single copy gene template (at this temperature there is no signal from the telomere PCR product, because it is fully melted).The qPCR instrument data analysis software was used for raw data analysis, in order to compare Ct with the reference sample. This comparison gives the T/S ratio, using the −ΔΔCt formula. The average amplification efficiency and coefficient of determination (R2) for the telomere sequence was 99.87% and 0.9993, and for the reference gene was 99.40% and 0.9992.

−ΔΔCt formula: $T/S = E_{tel}^{(Cttel\text{-}standard - Cttel\text{-}sample)} / E_{alb}^{(Ctalb\text{-}standard - Ct\ alb\text{-}sample)}$

where E_{tel} and E_{alb} are the amplification efficiency of the telomere and albumin sequence respectively, $C_t$tel-standard and $C_t$tel-sample are the Ct for the telomere sequence of the standard DNA and that of the sample respectively.

Each sample was measured in triplicate. Results with a replicate SD less than 10% were further analyzed (when SD >10%, samples were analyzed in the 4th PCR run). Triplicates for the same participant were run in the same assay plate to rule out potential plate-to-plate batch variation.

3. Questionnaires administered to participants

Assessment of children's perceived stress was made through "Stress in Children" (SiC) questionnaire [3]. It is a 21-question instrument designed for school-age children, aiming to assess perceived stress, levels of well-being, coping strategies and social support. It evaluates 3 components: the "degree of perceived distress (distress)" experienced by the child according to physical discomfort and emotional manifestations, the "lack of well-being" which refers to the levels of health and safety perceived by the child and the "lack of social support" which refers both to the behaviour of the people surrounding the child, as well as his own when he faces difficulties. Questions are on a Likert scale (1= Never, 2= Sometimes, 3= Often, 4= Very often) and participants are asked to answer how often they have felt or thought a certain way in the past month, with responses ranging from "not at all" to "very often," where high scores correspond to high levels of stress. This tool has been used in a Greek population [4]. Quality and lifestyle in children, health-related quality of life (HRQoL) has been measured in Greece with the weighted version [5] of the Pediatric Quality of Life Inventory™ (PedsQLT™) 4.0 Generic Core Scales [6]. The diabetes version of this tool (PedsQL™3.0 Diabetes Module) [7] has also been validated in a Greek pediatric population [8] to assess the quality of life of children with T1D, showing a lower diabetes-related quality of life for these children compared to their healthy peers. This questionnaire is completed either by the children (8 to 12 years old) or by the parent representative and is a psychological indicator of the quality of life of the child with diabetes, as defined by international medical recommendations. Responses are given on a 5-point Likert-type scale (from 0=never to 4=very often), with high scores indicating fewer problems, hence better Diabetes-Related Quality of Life. The PedsQL will normally also be completed by control group participants. Assessment of parent stress levels Perceived Stress Scale (PSS 14), weighted in Greek [9]. The Perceived Stress Scale (PSS) is a 14-item self-report instrument [10] that measures the degree to which situations in a person's life are appraised as stressful. Frequency of feelings and thoughts during the past month is rated on a 5-point Likert-type scale (from 0=never to 4=very often). There are seven positive and seven negative items, and the total score is calculated by summing the score of each item after all positive items have been reversed (minimum total score=0, maximum total score=56). Higher scores indicate the individual's higher level of perceived stress during the past month. Good psychometric properties of this measure have been reported in the Greek population [13]. Children above the age of 8 years old, completed the questionnaires alone without the guidance of their parents. An assistance was provided by the main

researcher to all children under the age of 8 and those feeling unable to comprehend the questions. All questionnaires were completed at the participant's visit at the hospital.

4. Supplementary Figures and Tables

4.1 Correlations of parameters in the T1D group

Table 1 depicts results of intercorrelations among the significant variables concerning children with T1D.

Table 1: Correlations among T/S ratio, clinical and psychosocial characteristics of children with T1D (n=35)

**Parameters<br>Correlation coefficient (r) / number of children (n)**												
**Parameters<br>r / n**	T/S ratio	Cort	HsCRP	HbA1c	BMI z-score	SiC total	Wellbeing	Social Support	Distress	PSS	PedsQL score	T1DM duration
T/S ratio	1	-	-	-	-	-	-	-	-	-	-	-
Cort	-0.083<br>32	1	-	-	-	-	-	-	-	-	-	-
HsCRP	0.208<br>35	0.116<br>32	1	-	-	-	-	-	-	-	-	-
HbA1c	0.189<br>35	-0.259<br>32	0.243<br>35	1	-	-	-	-	-	-	-	-
BMI z-score	0.157<br>35	-0.231<br>32	**0.407***<br>35	0.258<br>35	1	-	-	-	-	-	-	-
SiC total	0.333<br>35	0.219<br>32	0.179<br>35	0.101<br>35	-0.076<br>35	1	-	-	-	-	-	-
Wellbeing	0.259<br>35	0.059<br>32	0.205<br>35	0.043<br>35	0.010<br>35	**0.502****<br>35	1	-	-	-	-	-
Social Support	**0.347***<br>35	0.214<br>32	0.239<br>35	0.047<br>35	0.050<br>35	**0.817****<br>35	0.307<br>35	1	-	-	-	-
Distress	0.070<br>35	0.250<br>32	0.140<br>35	0.142<br>35	0.026<br>35	**0.559****<br>35	**0.420***<br>35	0.293<br>35	1	-	-	-
PSS	0.189<br>35	-0.073<br>32	0.188<br>35	0.123<br>35	0.074<br>35	0.085<br>35	0.157<br>35	-0.139<br>35	0.155<br>35	1	-	-
PedsQL score	-0.152<br>35	0.211<br>32	-0.108<br>35	**-0.408***<br>35	0.024<br>35	-0.319<br>35	-0.163<br>35	-0.191<br>35	**-0.338***<br>35	-0.256<br>35	1	-
T1DM duration	0.137<br>35	**-0.355***<br>32	**-0.339***<br>35	0.205<br>35	0.085<br>35	-0.006<br>35	-0.159<br>35	0.077<br>35	-0.039<br>35	-0.122<br>35	-0.207<br>35	1

*$p < 0.05$, **$p < 0.001$. Pearson or Spearman Rho. Bold stands for statistical significance.

r= Correlation Coefficient, T/S= Telomere/Single copy gene ratio, T1DM = Type 1 Diabetes Mellitus, Cort= Serum Cortisol, HsCRP= High Sensitivity CRP, HbA1c= Glycated Hemoglobin, BMI= Body Mass Index, PSS= Perceived Stress Scale, SiC= Stress in Children, PedsQL= Paediatric Scale Quality of Life. Wellbeing, Social Support, and Distress = estimated units of Stress in Children Questionnaire.

4.2 Alternative multivariate regression analysis models in the T1D group

In the following Table alternative multivariate regression analysis models are presented, concerning the T1D group. Gender is the only independent variable that remains important as a covariate in rLTL assessment. Gender X social support is significant (p= 0.007), or borderline significant (p= 0.051).

Table 2: Regression analysis on parameters related to T1D children's rLTL (n=35).

	**Coefficients[a]**						
**Independent Variables[b]**	**Unst. Coeff**		**Stand Coeff**	**t**	**Sig.**	**Collinearity Statistics**	
	B	SE	Beta			Tol	VIF
**Model 1**							
Child's age	0.003	0.010	0.047	0.311	0.758	0.956	1.046
Child's gender	-0.391	0.107	-1.448	-3.657	**< 0.001**	0.137	7.300
Gender X Social support	0.099	0.034	1.150	2.910	**0.007**	0.137	7.275
T1D duration	0.017	0.011	0.242	1.615	0.117	0.956	1.046
**Model 2**							
Child's age	0.001	0.010	0.022	0.141	0.889	0.899	1.112
Child's gender	-0.502	0.203	-1.859	-2.469	**0.020**	0.039	25.895
Gender X Social support	0.136	0.067	1.580	2.032	0.051	0.036	27.629
Social support	-0.038	0.058	-0.193	-0.644	0.524	0.245	4.082
T1D duration	0.019	0.011	0.269	1.713	0.097	0.888	1.126

[a] Dependent Variable: Relative LTL (lymphocyte telomere length)

[b] Covariates: gender, age, diabetes duration, parental age. Sex: 0= female, 1= male.

Regression analysis in three consecutive models.

Model 1: R square = 0.356, $p = 0.009$, F = 4.145.

Model 2: R square = 0.365, $p = 0.017$, F = 3.334.

* means statistical significance.

Statistical significance level at $p < 0.05$. Bold stands for statistical significance.

4.3 Multivariate regression analysis models in the healthy control group

Multivariate regression analysis in the Healthy Control group ran separate in 3 models. The third model included the following covariates: child's age, gender, social support, parental age, educational level, occupational status and marital status of the caregiver parent. Parents were by 68.6% the same caregivers with the T1DM group.

Table 3: Regression analysis on parameters related to healthy control's rLTL (n=35).

	Coefficients[a]						
**Independent Variables[b]**	**Unst. Coeff**		**Stand Coeff**	**t**	**Sig.**	**Collinearity Statistics**	
	B	SE	Beta			Tol	VIF
**Model 1**	**Basic model**						
Child gender	-0.041	0.039	-0.188	-1.044	0.305	0.845	1.183
Child age	0.005	0.012	0.080	0.421	0.677	0.761	1.314
Social support	-0.047	0.038	-0.309	-1.734	0.093	0.865	1.156
**Model 2**	**Extended model**						
Child gender	-0.040	0.040	-0.185	-1.006	0.322	0.841	1.189
Child age	0.005	0.012	0.080	0.414	0.682	0.761	1.314
Parental age	-0.001	0.003	-0.045	-0.256	0.800	0.919	1.088
Social support	-0.069	0.040	0.321	-1.716	0.097	0.806	1.240
**Model 3**	**Sociodemographic model**						
Child gender	-0.044	0.043	-0.203	-1.037	0.309	0.774	1.291
Child age	0.007	0.012	0.109	0.531	0.600	0.701	1.426
Parental age	-0.001	0.004	-0.073	-0.402	0.690	0.894	1.118
Social support	-0.092	0.046	-0.425	-1.983	0.058	0.646	1.547
Parent occupation	-0.052	0.061	-0.168	-0.849	0.403	0.756	1.323
Parent education	0.034	0.042	0.159	0.819	0.420	0.786	1.272
Marital status	0.048	0.099	0.102	0.483	0.633	0.664	1.506

[a] Dependent Variable: Relative LTL (lymphocyte telomere length)

[b] Covariates: Independent Variables concerning children's and parental/caregiver characteristics in 3 consecutive models. Gender: 0= female, 1= male.

Regression analysis in three consecutive models.

Model 1: R square = 0.149, $p = 0.166$, F= 1.809.

Model 2: R square = 0.151, $p = 0.281$, F = 1.333.

Model 3: R square = 0.198, $p = 0.483$, F= 0.954

Statistical significance level at $p < 0.05$.

Supplementary References

1. Hsieh, A.Y.; Saberi, S.; Ajaykumar, A.; Hukezalie, K.; Gadawski, I.; Sattha, B.; Côté, H.C. Optimization of a Relative Telomere Length Assay by Monochromatic Multiplex Real-Time Quantitative PCR on the LightCycler 480: Sources of Variability and Quality Control Considerations. *J. Mol. Diagn.* **2016**, *18*, 425–437. https://doi.org/10.1016/j.jmoldx.2016.01.004.
2. Cawthon, R.M. Telomere measurement by quantitative PCR. *Nucleic Acids Res.* **2002**, *30*, e47. https://doi.org/10.1093/nar/30.10.e47.
3. Osika, W.; Friberg, P.; Wahrborg, P. A new short self-rating questionnaire to assess stress in children. *Int. J. Behav. Med.* **2007**, *14*, 108–117. https://doi.org/10.1007/BF03004176.
4. Emmanouil, C.C.; Pervanidou, P.; Charmandari, E.; Darviri, C.; Chrousos, G.P. The effectiveness of a health promotion and stress-management intervention program in a sample of obese children and adolescents. *Hormones* **2018**, *17*, 405–413. https://doi.org/10.1007/s42000-018-0052-2.
5. Gkoltsiou, K.; Dimitrakaki, C.; Tzavara, C.; Papaevangelou, V.; Varni, J.W.; Tountas, Y. Measuring health-related quality of life in Greek children: Psychometric properties of the Greek version of the Pediatric Quality of Life Inventory(TM) 4.0 Generic Core Scales. *Qual. Life Res.* **2008**, *17*, 299–305. https://doi.org/10.1007/s11136-007-9294-1.
6. Varni, J.W.; Seid, M.; Kurtin, P.S.; PedsQL 4.0: reliability and validity of the Pediatric Quality of Life Inventory version 4.0 generic core scales in healthy and patient populations. *Med Care* **2001**, *39*(8), 800-812. https://doi.org/10.1097/00005650-200108000-00006.
7. Varni, J.W.; Burwinkle, T.M.; Jacobs, J.R.; Gottschalk, M.; Kaufman, F.; Jones, K.L. The PedsQL in type 1 and type 2 diabetes: Reliability and validity of the Pediatric Quality of Life Inventory Generic Core Scales and type 1 Diabetes Module. *Diabetes Care* **2003**, *26*, 631–637. https://doi.org/10.2337/diacare.26.3.631.
8. Emmanouilidou, E.; Galli-Tsinopoulou, A.; Karavatos, A.; Nousia-Arvanitakis, S. Quality of life of children and adolescents with diabetes of Northern Greek origin. *Hippokratia* **2008**, *12*, 168–175.
9. Andreou, E.; Alexopoulos, E.C.; Lionis, C.; Varvogli, L.; Gnardellis, C.; Chrousos, G.P.; Darviri, C. Perceived Stress Scale: Reliability and validity study in Greece. *Int. J. Environ. Res. Public Health* **2011**, *8*, 3287–3298. https://doi.org/10.3390/ijerph8083287.
10. Cohen, S.; Kamarck, T.; Mermelstein, R.; A Global Measure of Perceived Stress. Journal of Health and Social Behavior. *Journal of health and social behavior* **1983**, *24*(4), 385–396.
