# Supplementary figures and images for: Relative Leukocyte Telomere Length Is Shorter in Children and Adolescents with Type 1 Diabetes: Screening of Basic Psychosocial Aspects
Source: Int J Mol Sci. 2026 Apr 27;27(9):3895. doi: 10.3390/ijms27093895 (PMC13164480; doi:10.3390/ijms27093895)

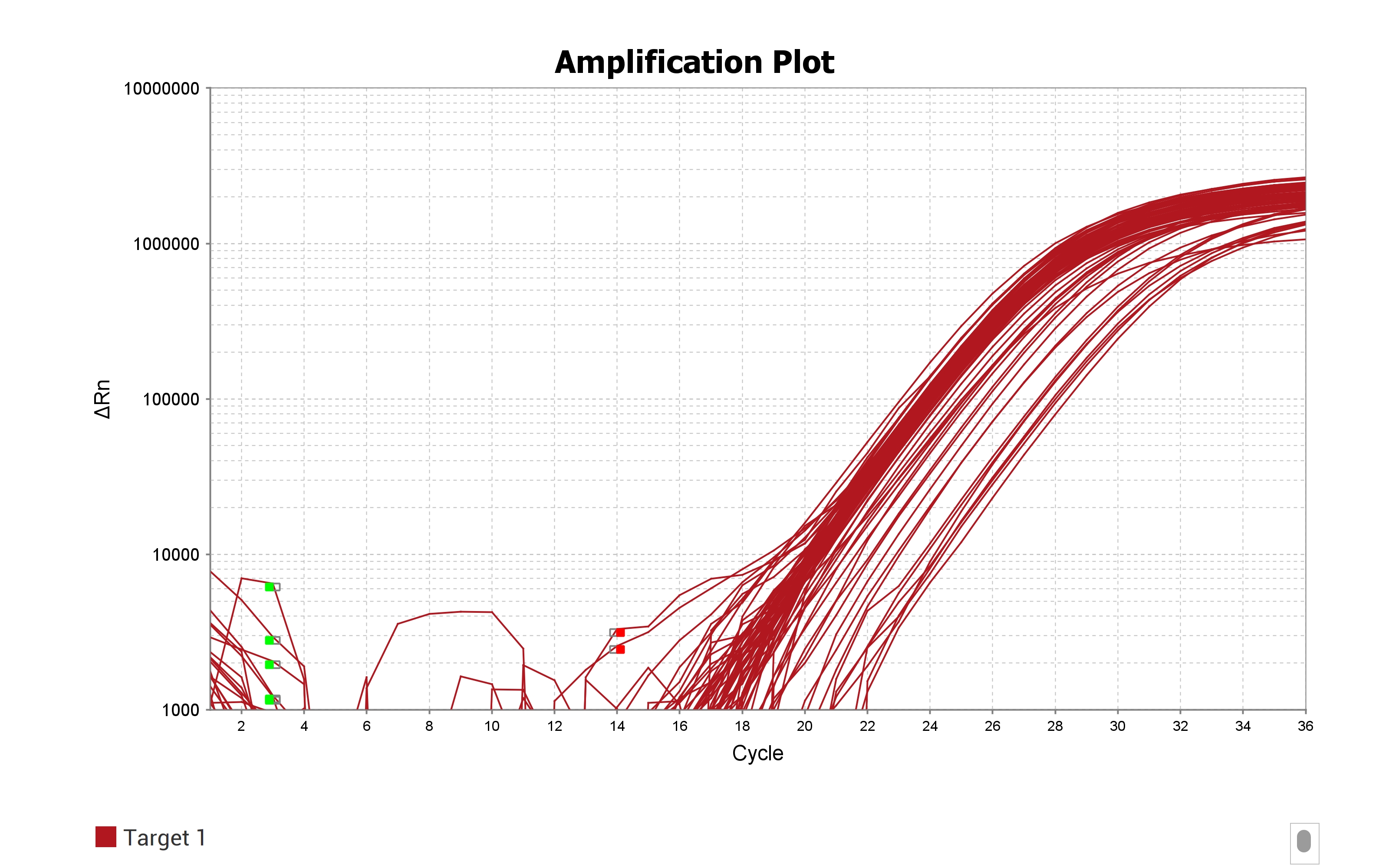

Supplement: Supplementary file 1 [file ijms-27-03895-s001.zip › pcrgraphs/Amplification Plot 13.12.23.jpg]

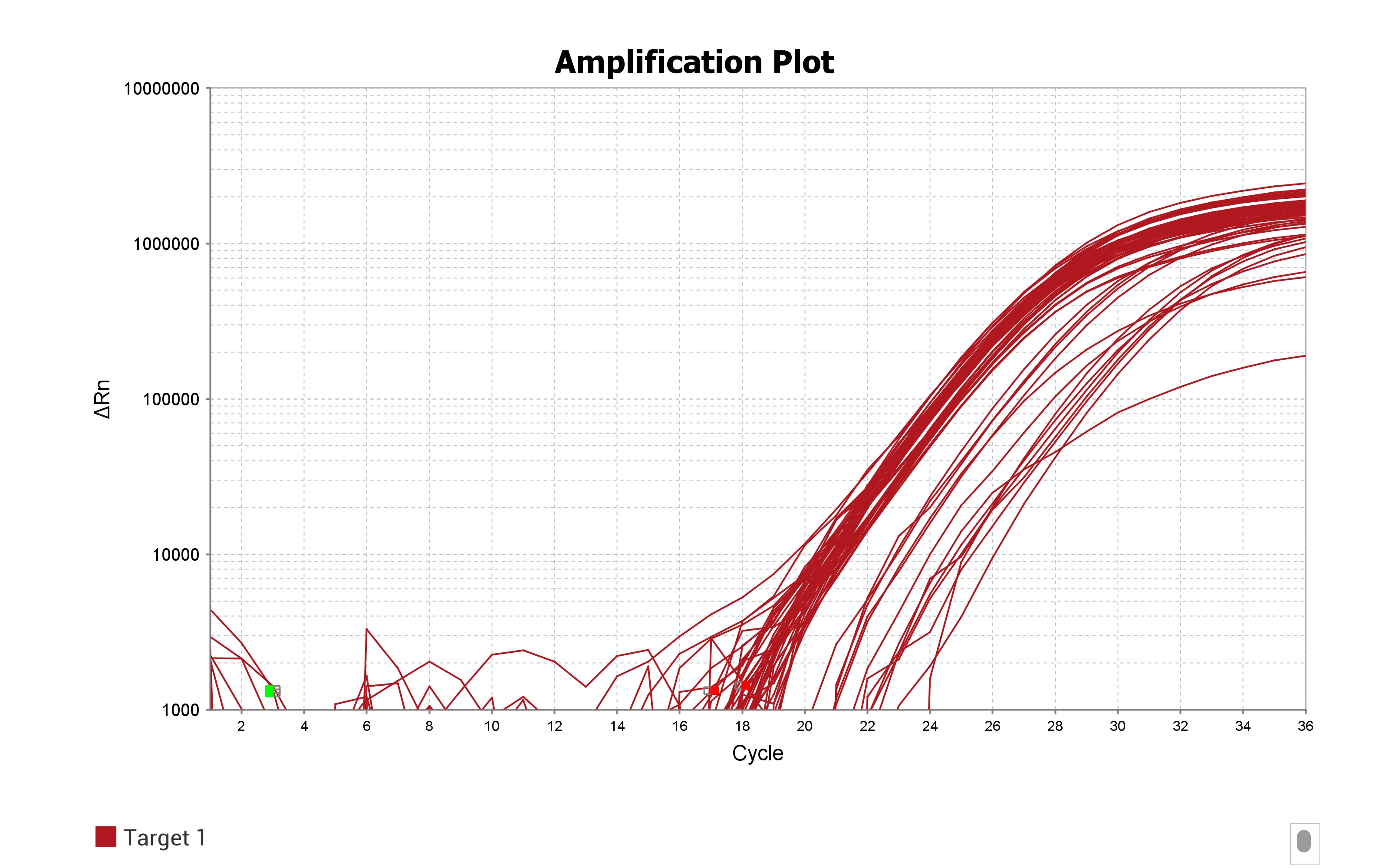

Supplement: Supplementary file 1 [file ijms-27-03895-s001.zip › pcrgraphs/Amplification Plot 16.2.24 2nd.jpg]

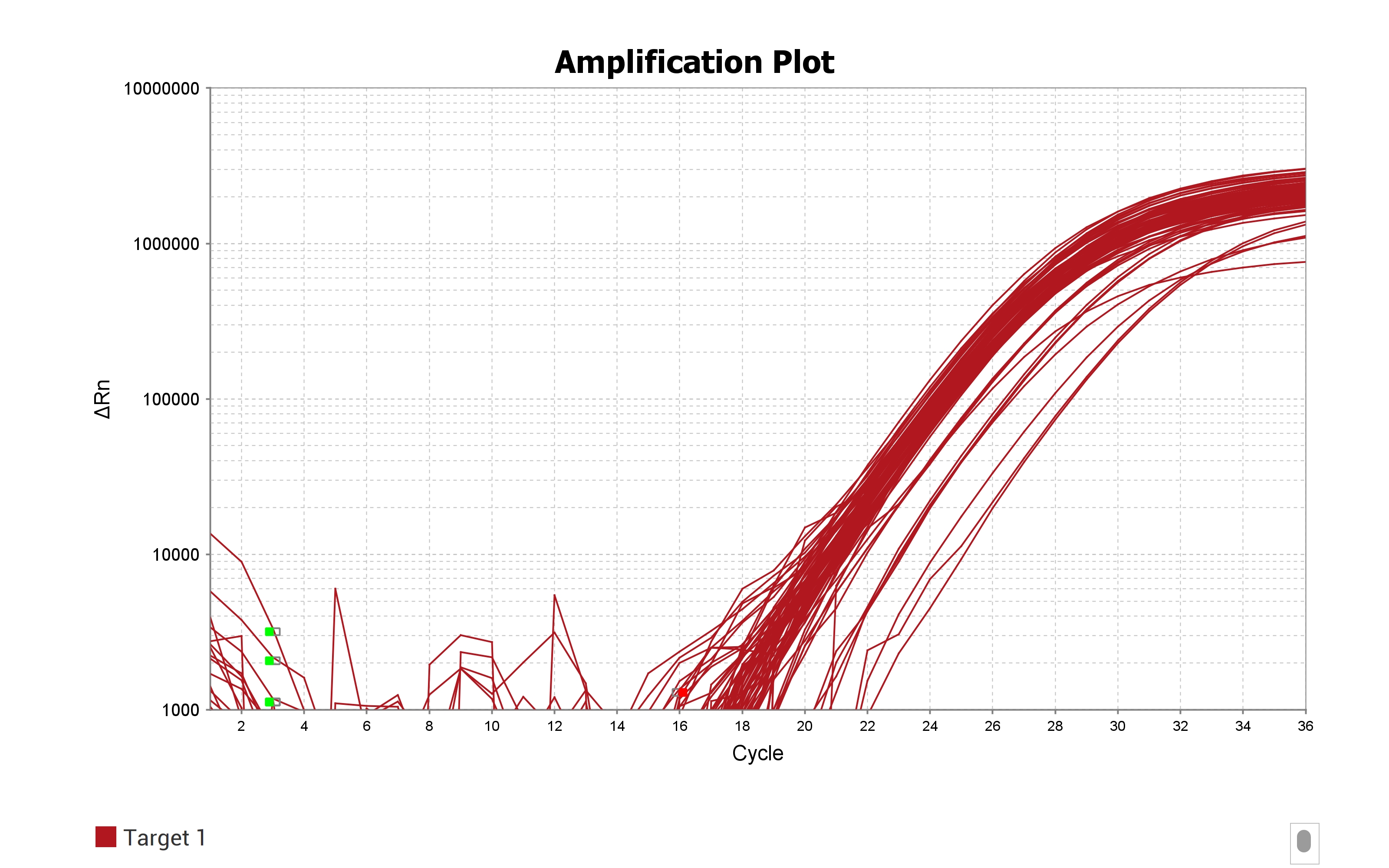

Supplement: Supplementary file 1 [file ijms-27-03895-s001.zip › pcrgraphs/Amplification Plot 16.2.24.jpg]

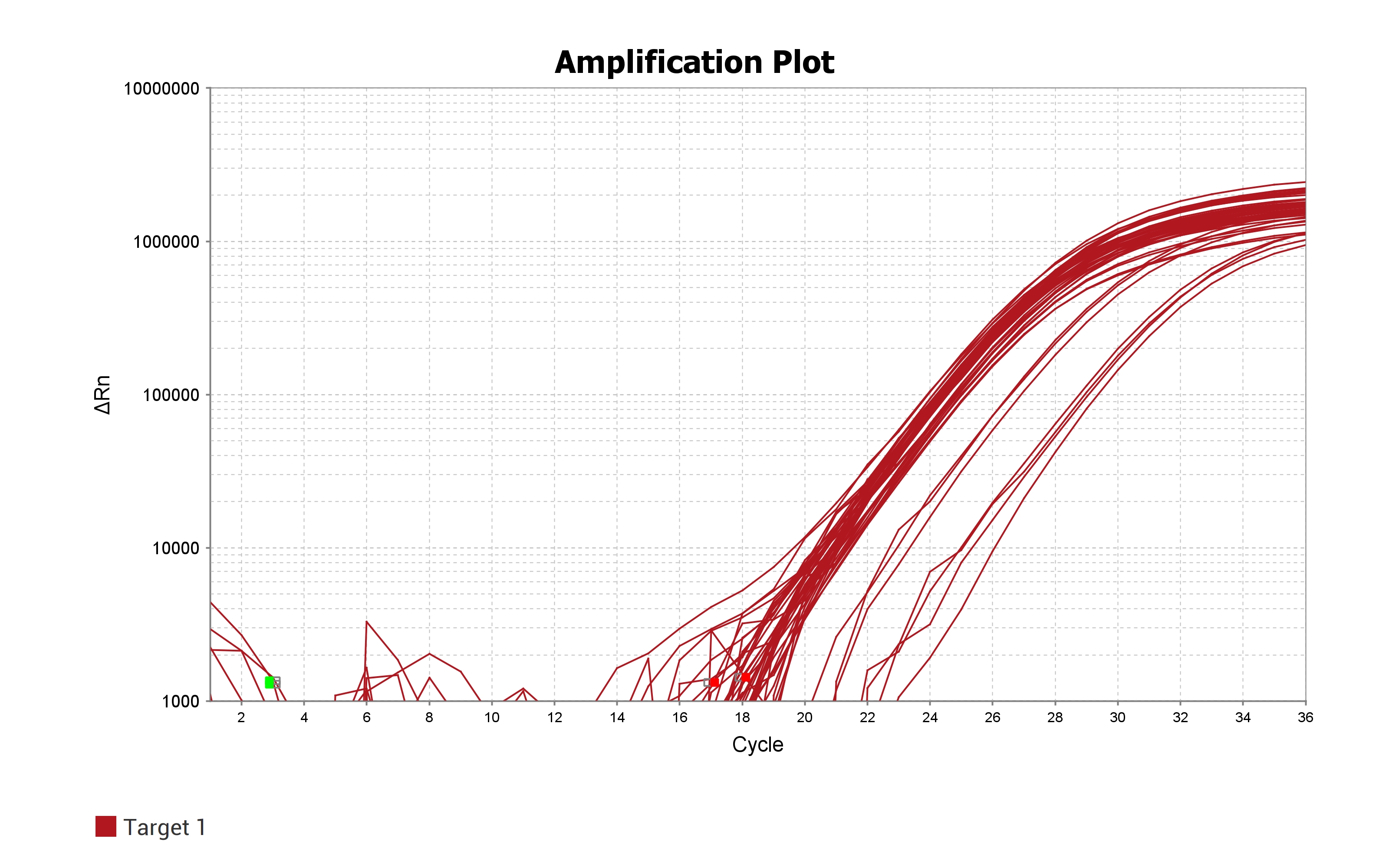

Supplement: Supplementary file 1 [file ijms-27-03895-s001.zip › pcrgraphs/Amplification Plot 27.2.24.jpg]

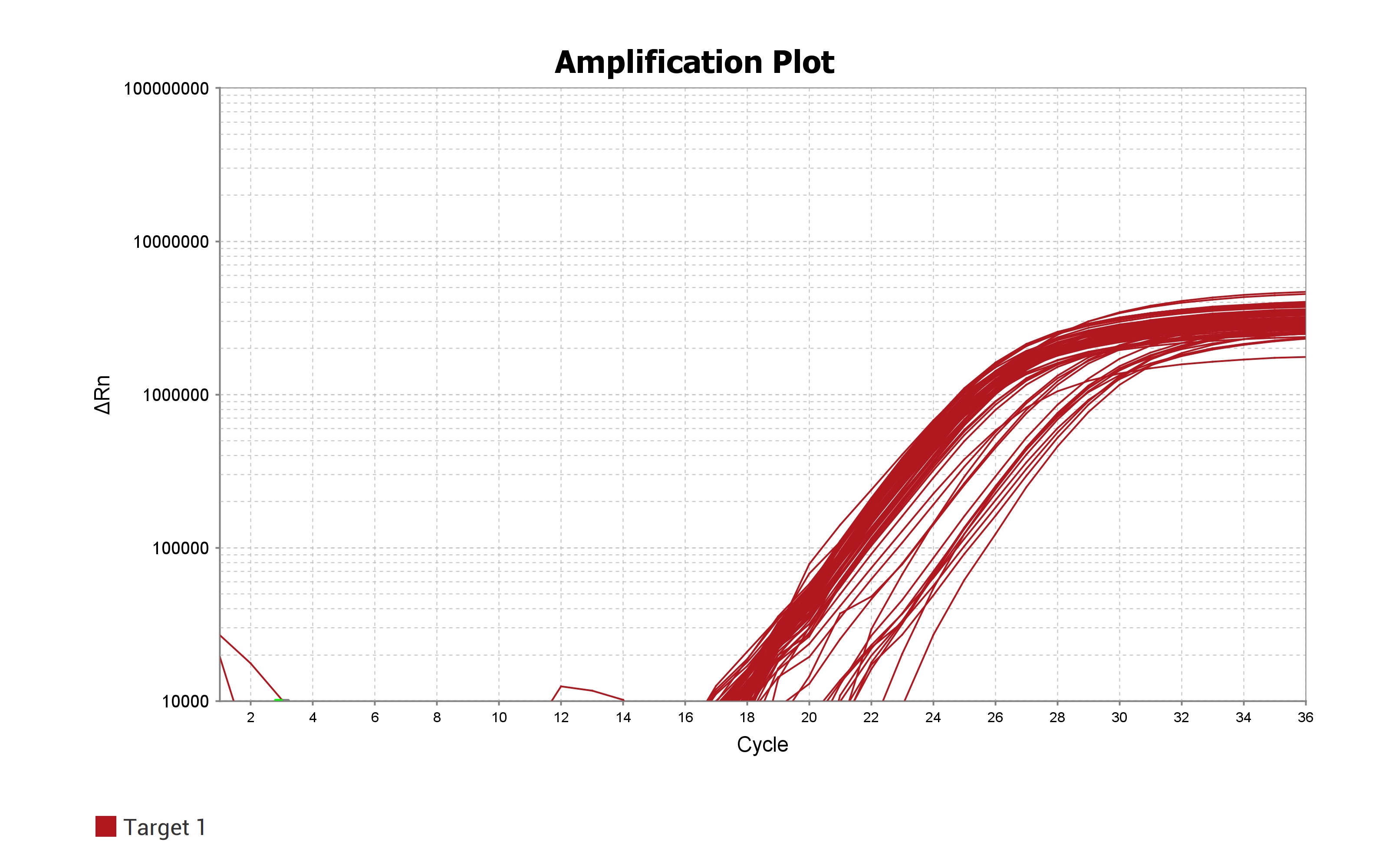

Supplement: Supplementary file 1 [file ijms-27-03895-s001.zip › pcrgraphs/Amplification Plot alb 13.12.23.jpg]

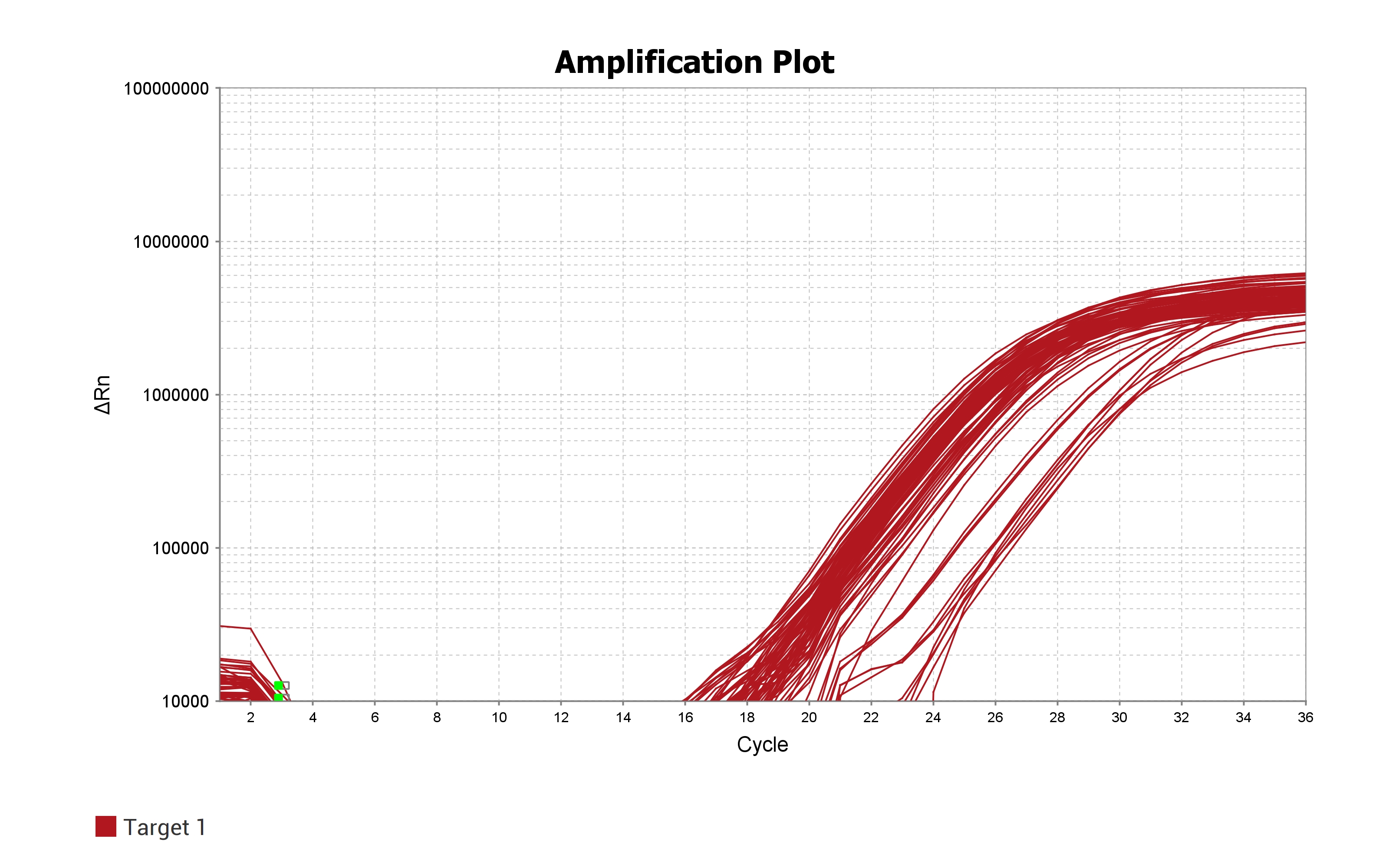

Supplement: Supplementary file 1 [file ijms-27-03895-s001.zip › pcrgraphs/Amplification Plot alb 16.2.24 2nd.jpg]

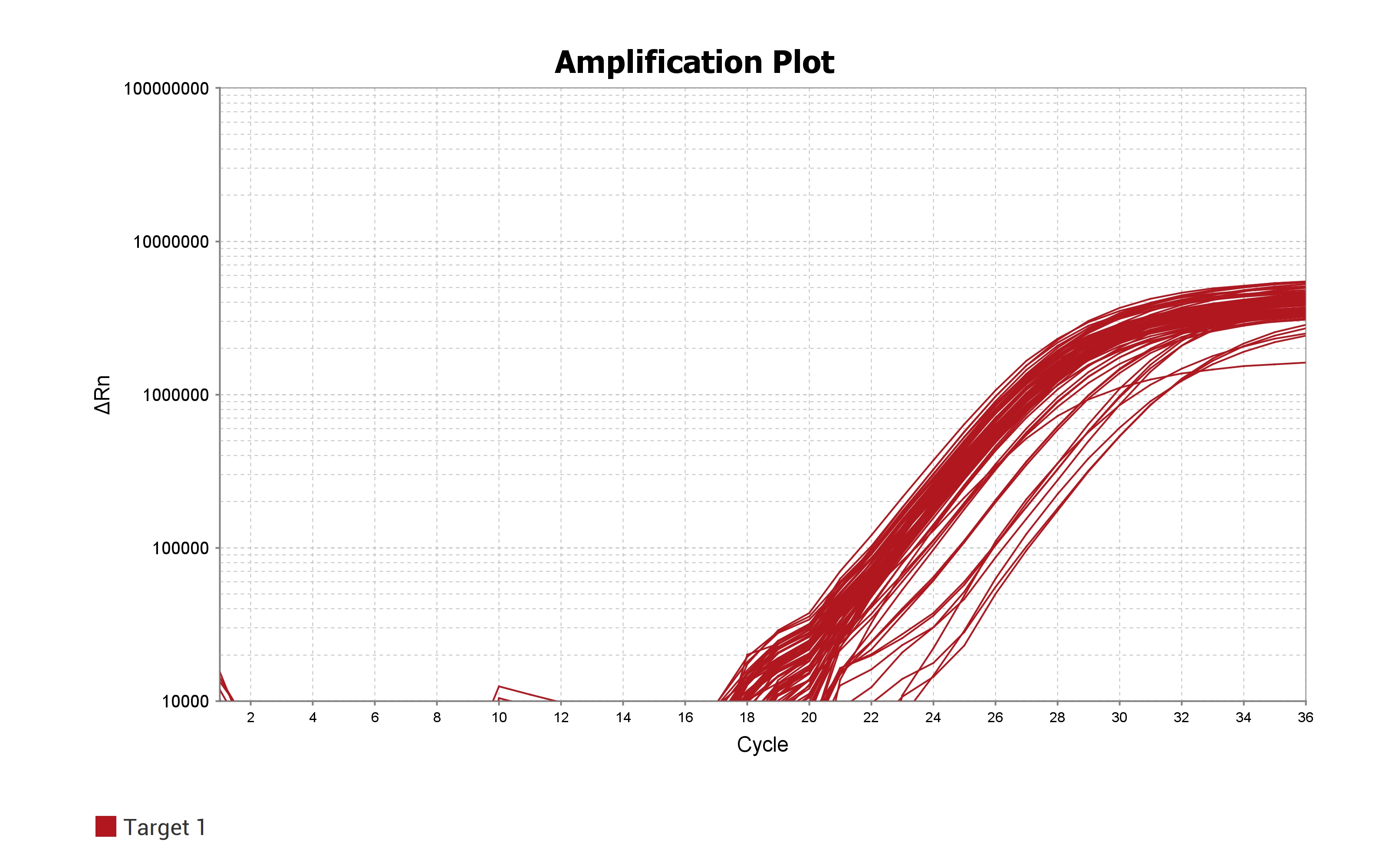

Supplement: Supplementary file 1 [file ijms-27-03895-s001.zip › pcrgraphs/Amplification Plot alb 16.2.24.jpg]

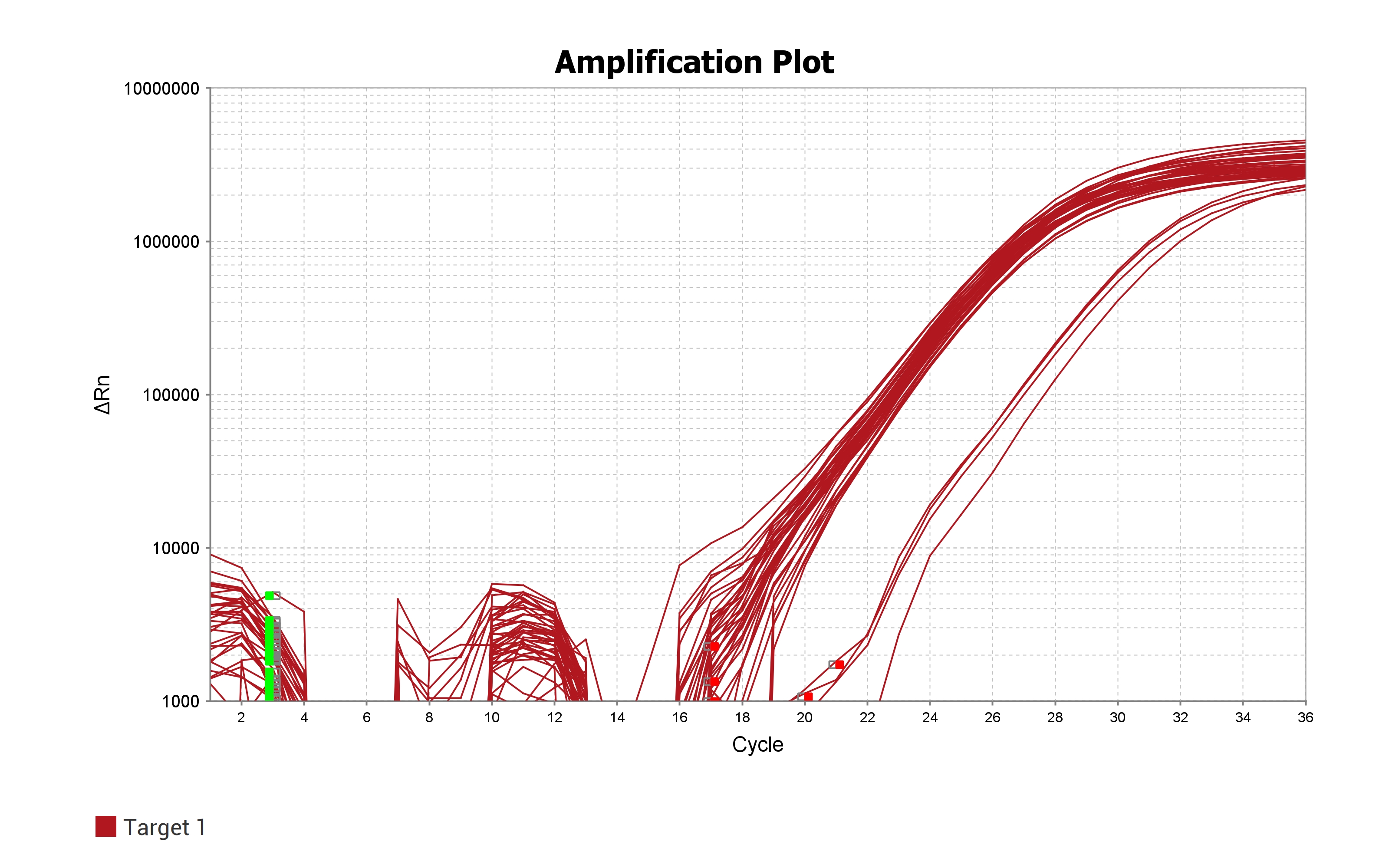

Supplement: Supplementary file 1 [file ijms-27-03895-s001.zip › pcrgraphs/Amplification Plot alb 27.2.24.jpg]

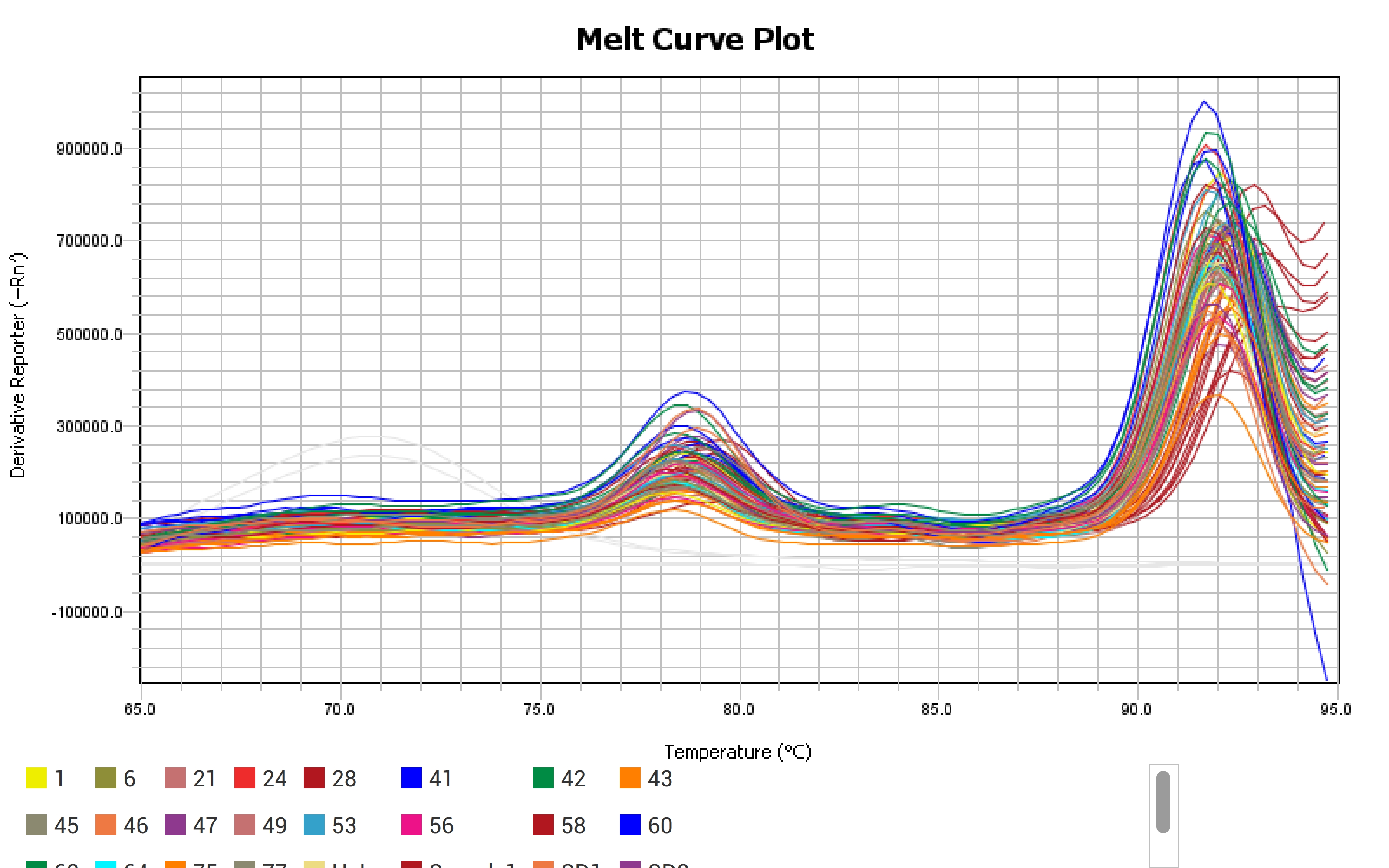

Supplement: Supplementary file 1 [file ijms-27-03895-s001.zip › pcrgraphs/Melt Curve Plot alb 13.12.23.jpg]

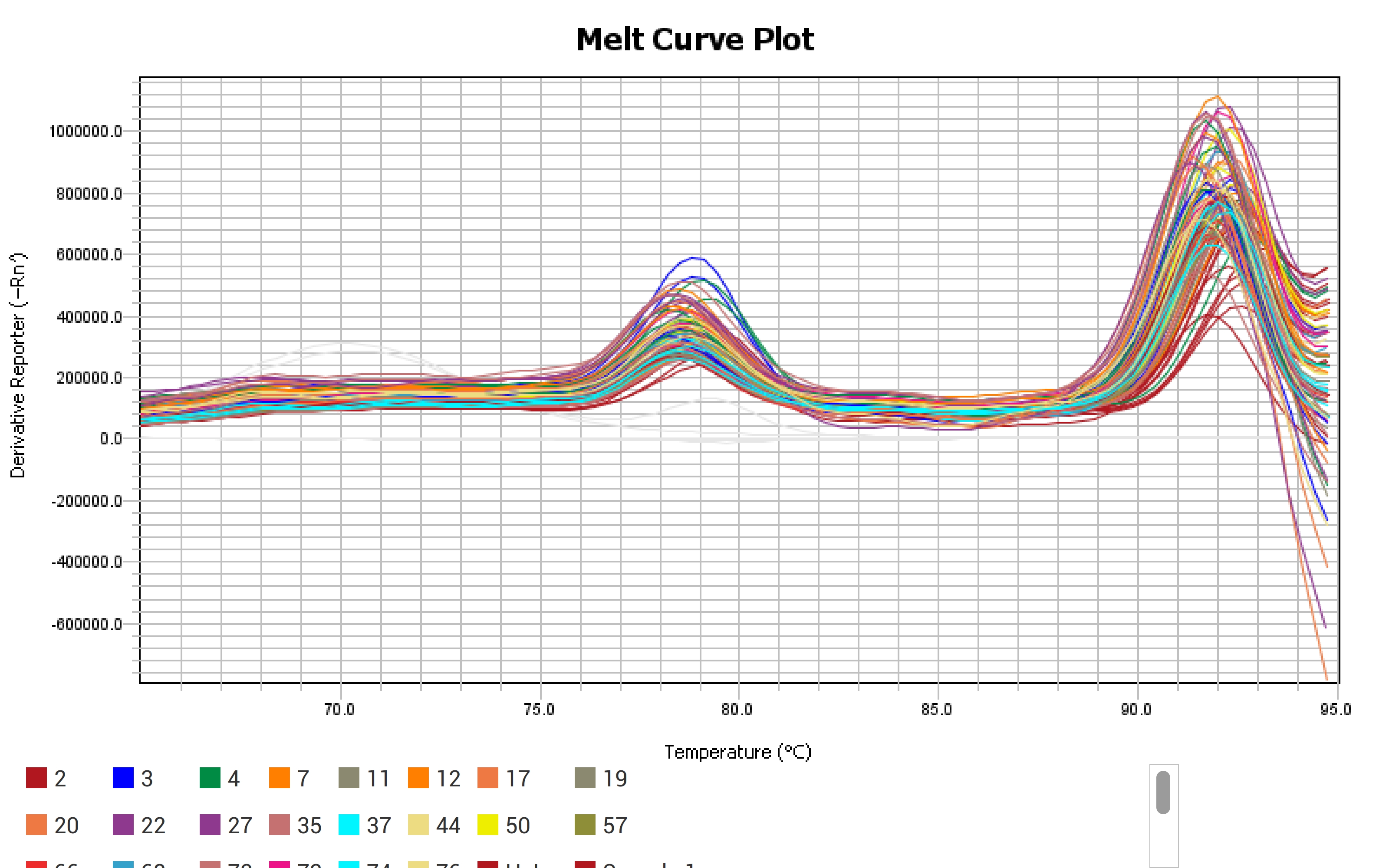

Supplement: Supplementary file 1 [file ijms-27-03895-s001.zip › pcrgraphs/Melt Curve Plot alb 16.2.24 2nd.jpg]

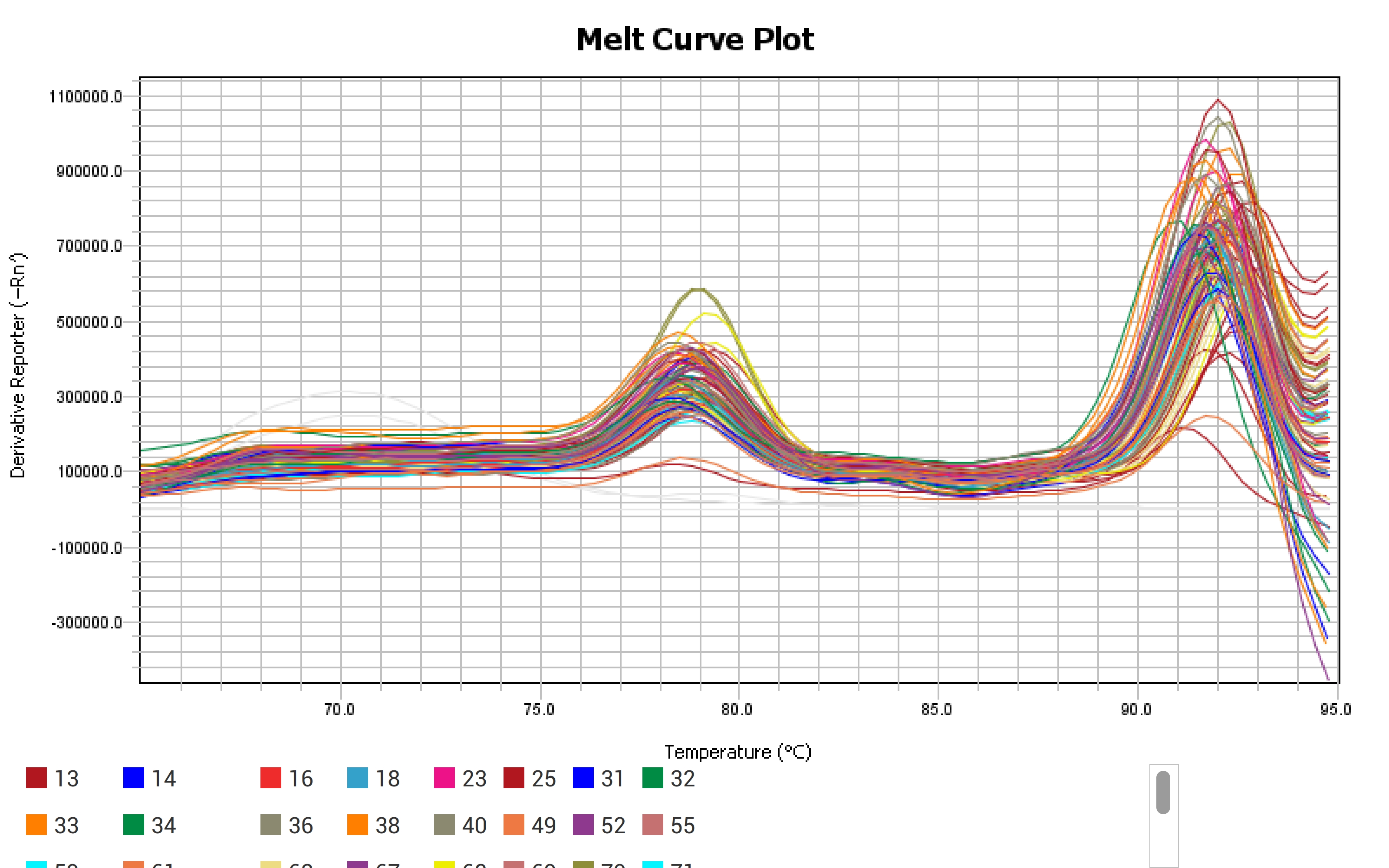

Supplement: Supplementary file 1 [file ijms-27-03895-s001.zip › pcrgraphs/Melt Curve Plot alb 16.2.24.jpg]

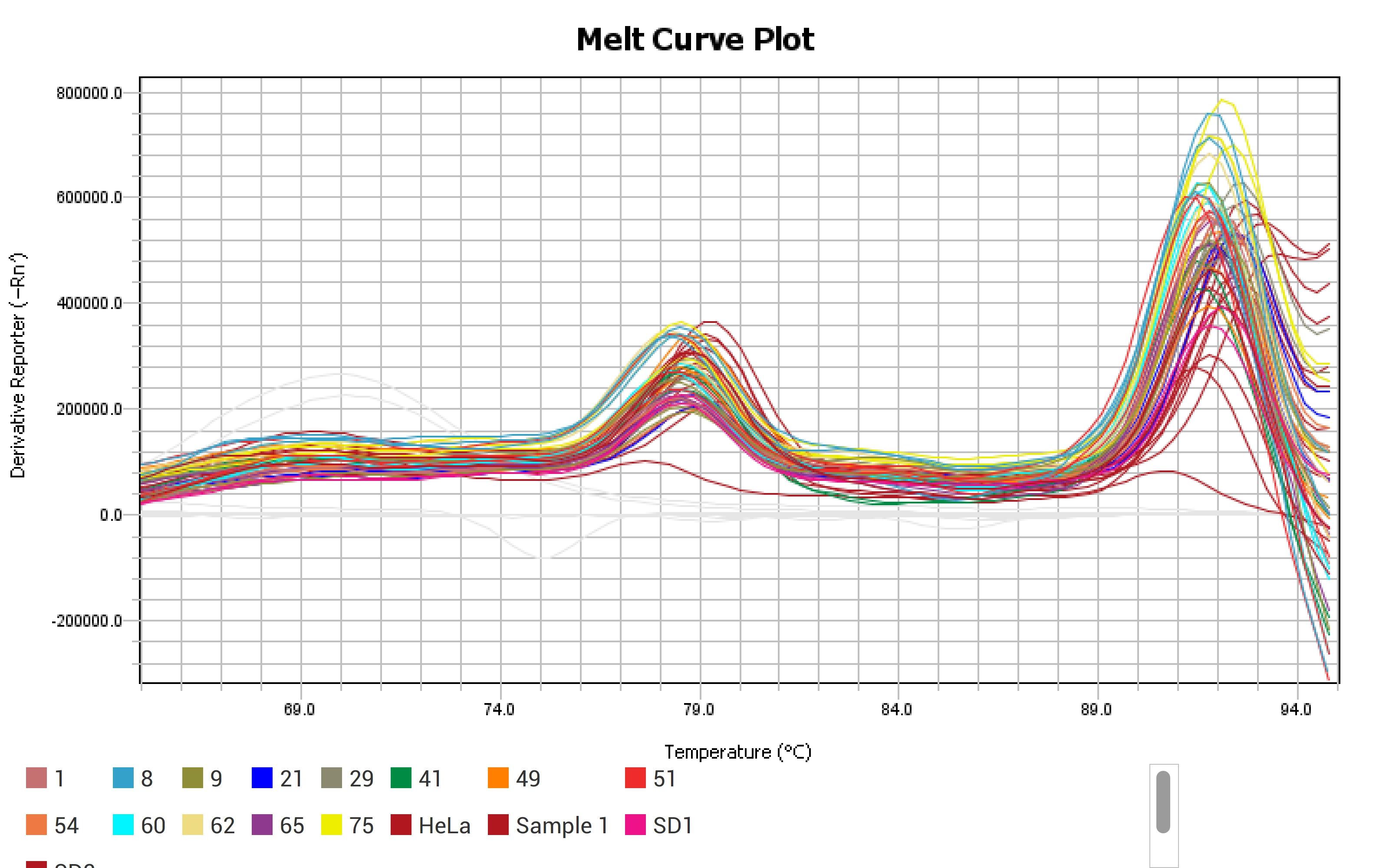

Supplement: Supplementary file 1 [file ijms-27-03895-s001.zip › pcrgraphs/Melt Curve Plot alb 27.2.24.jpg]

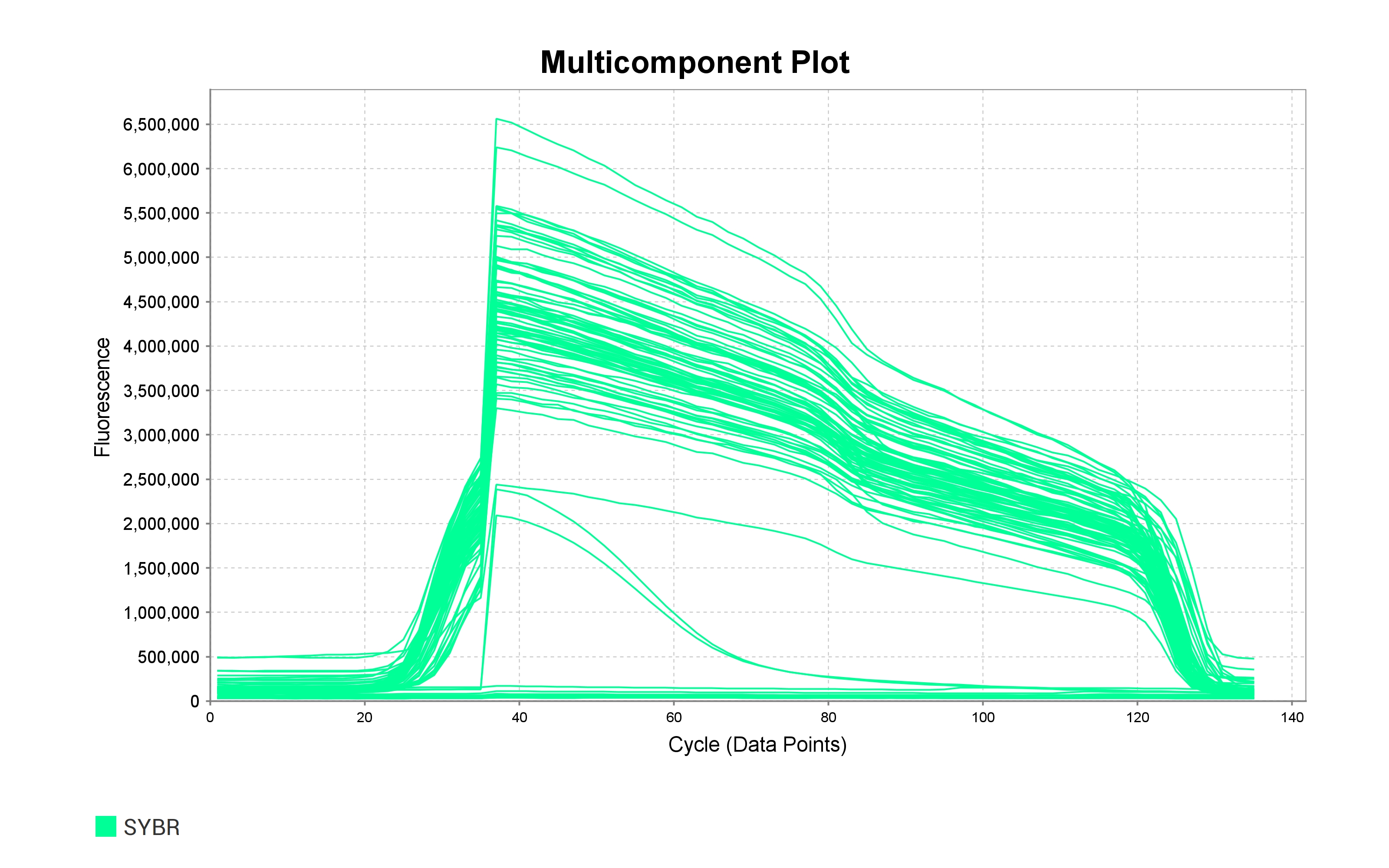

Supplement: Supplementary file 1 [file ijms-27-03895-s001.zip › pcrgraphs/Multicomponent Plot 13.12.23.jpg]

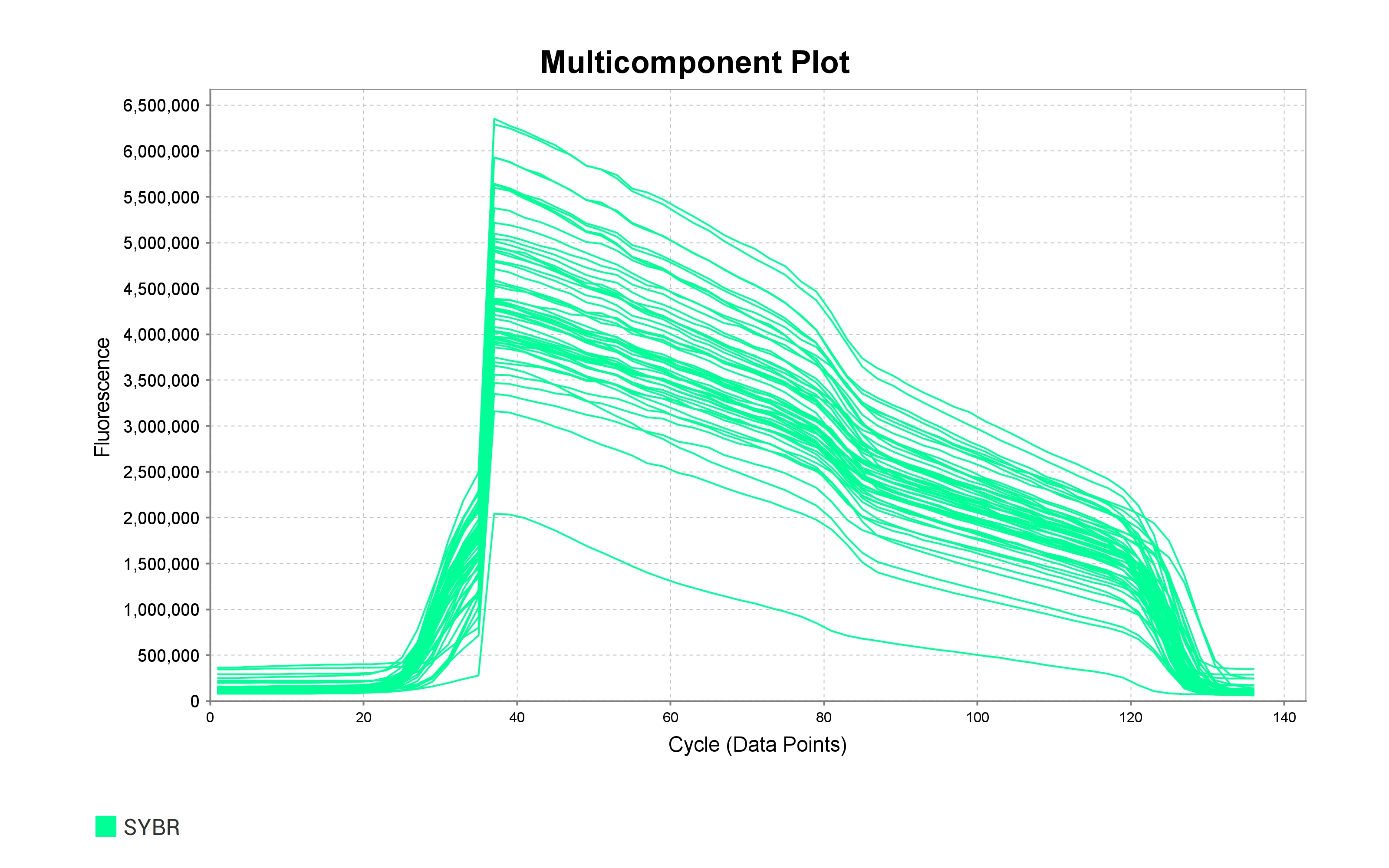

Supplement: Supplementary file 1 [file ijms-27-03895-s001.zip › pcrgraphs/Multicomponent Plot 16.2.24 2nd.jpg]

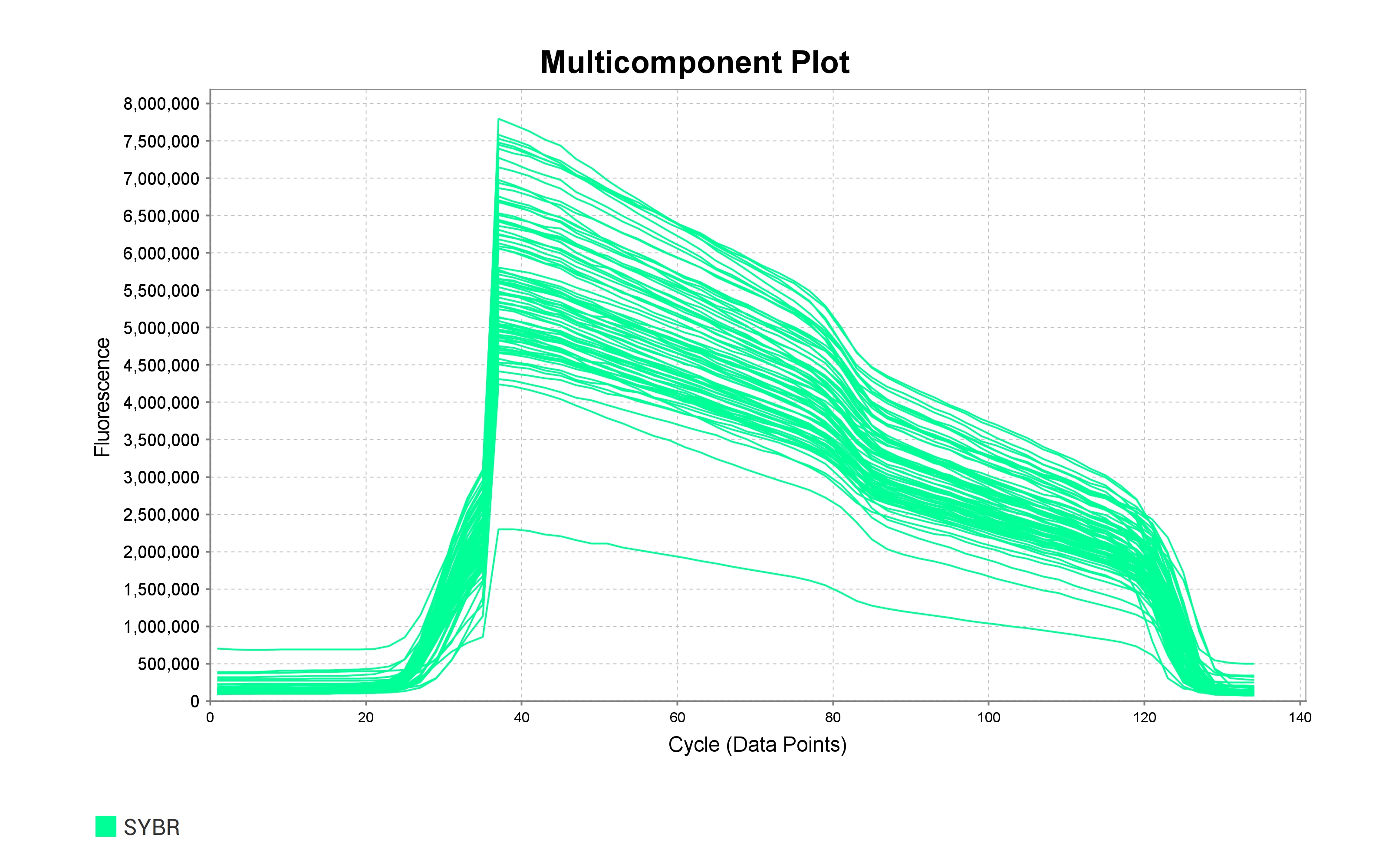

Supplement: Supplementary file 1 [file ijms-27-03895-s001.zip › pcrgraphs/Multicomponent Plot 16.2.24.jpg]

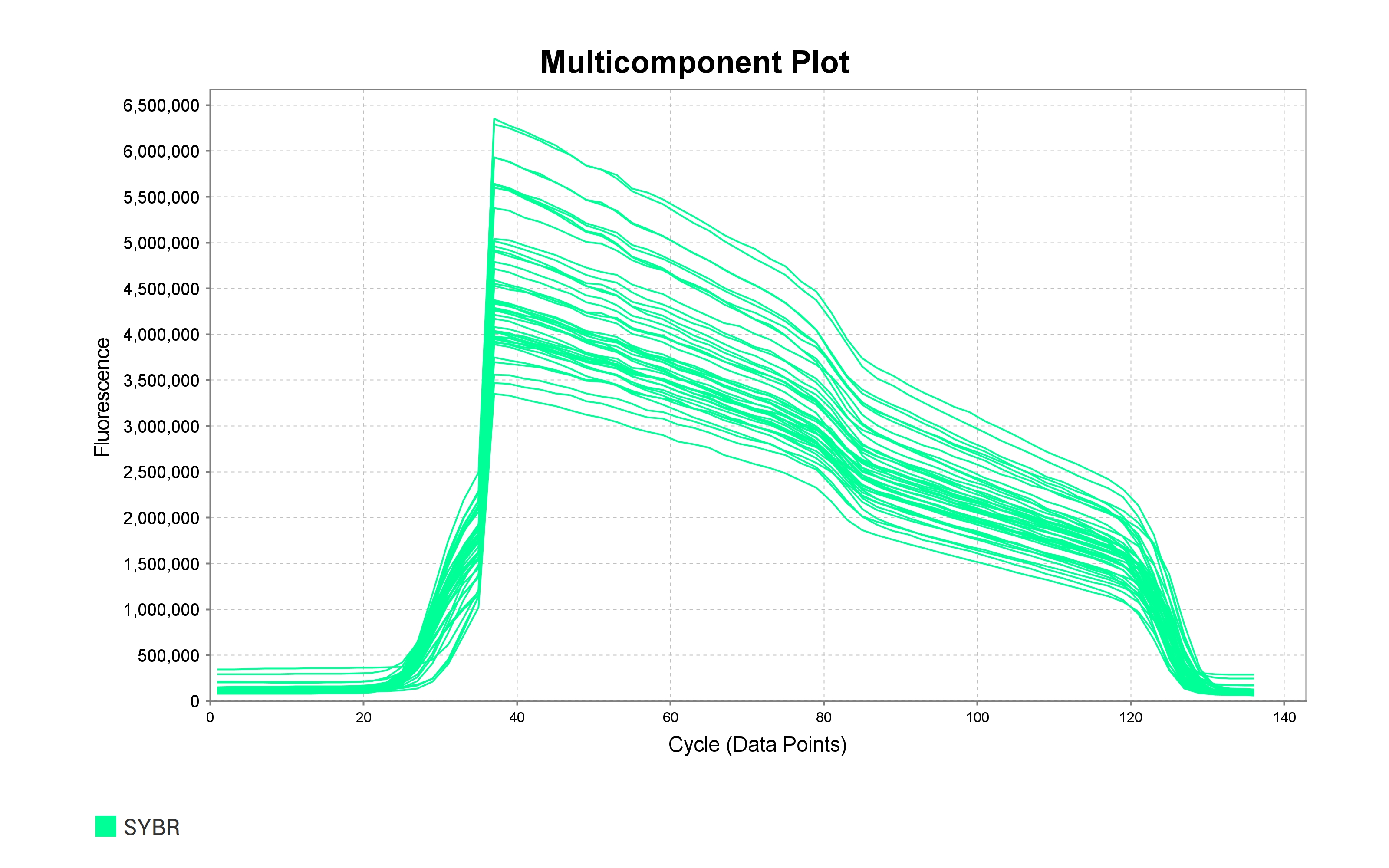

Supplement: Supplementary file 1 [file ijms-27-03895-s001.zip › pcrgraphs/Multicomponent Plot 27.2.24.jpg]

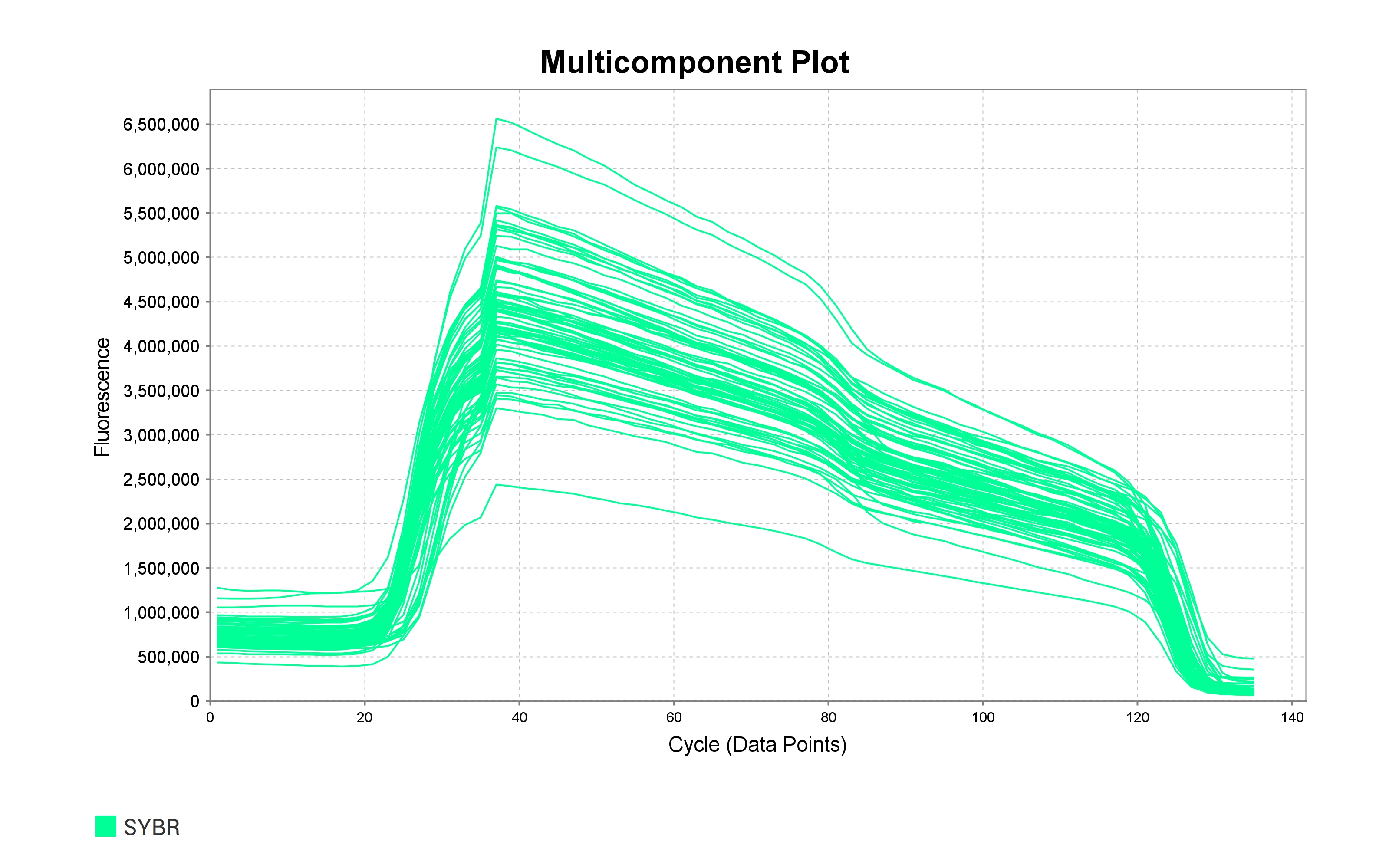

Supplement: Supplementary file 1 [file ijms-27-03895-s001.zip › pcrgraphs/Multicomponent Plot alb 13.12.23.jpg]

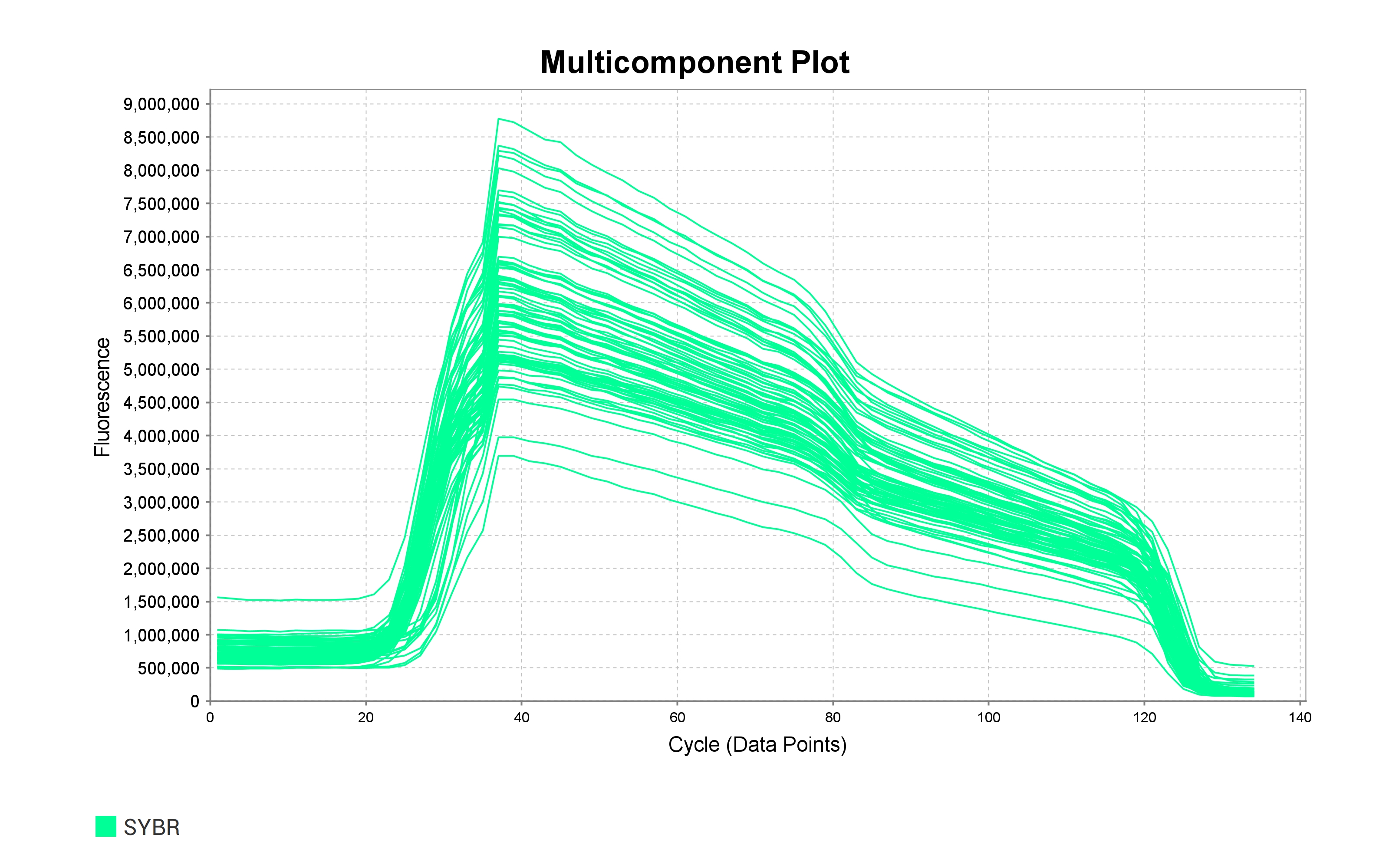

Supplement: Supplementary file 1 [file ijms-27-03895-s001.zip › pcrgraphs/Multicomponent Plot alb 16.2.24 2nd.jpg]

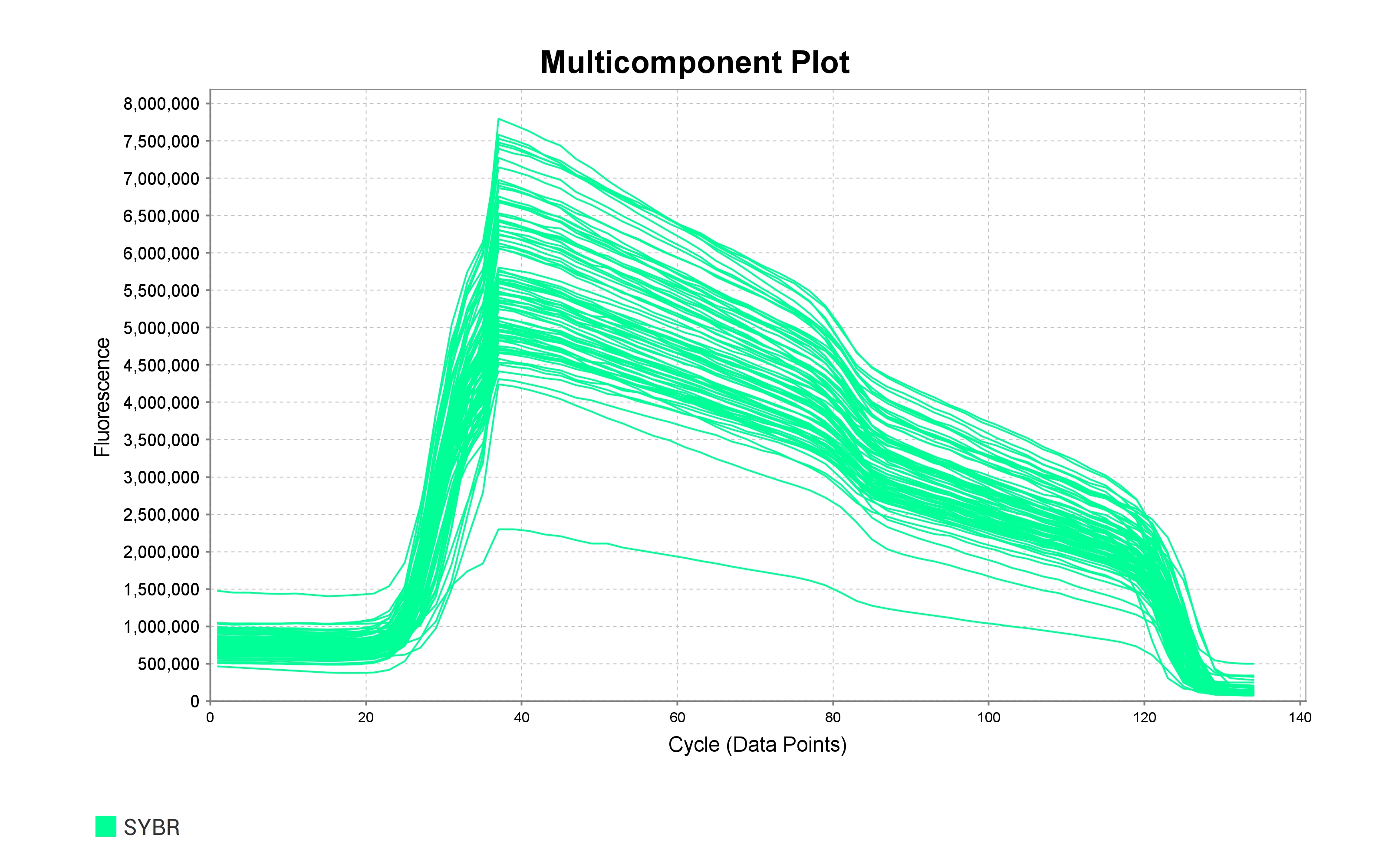

Supplement: Supplementary file 1 [file ijms-27-03895-s001.zip › pcrgraphs/Multicomponent Plot alb 16.2.24.jpg]

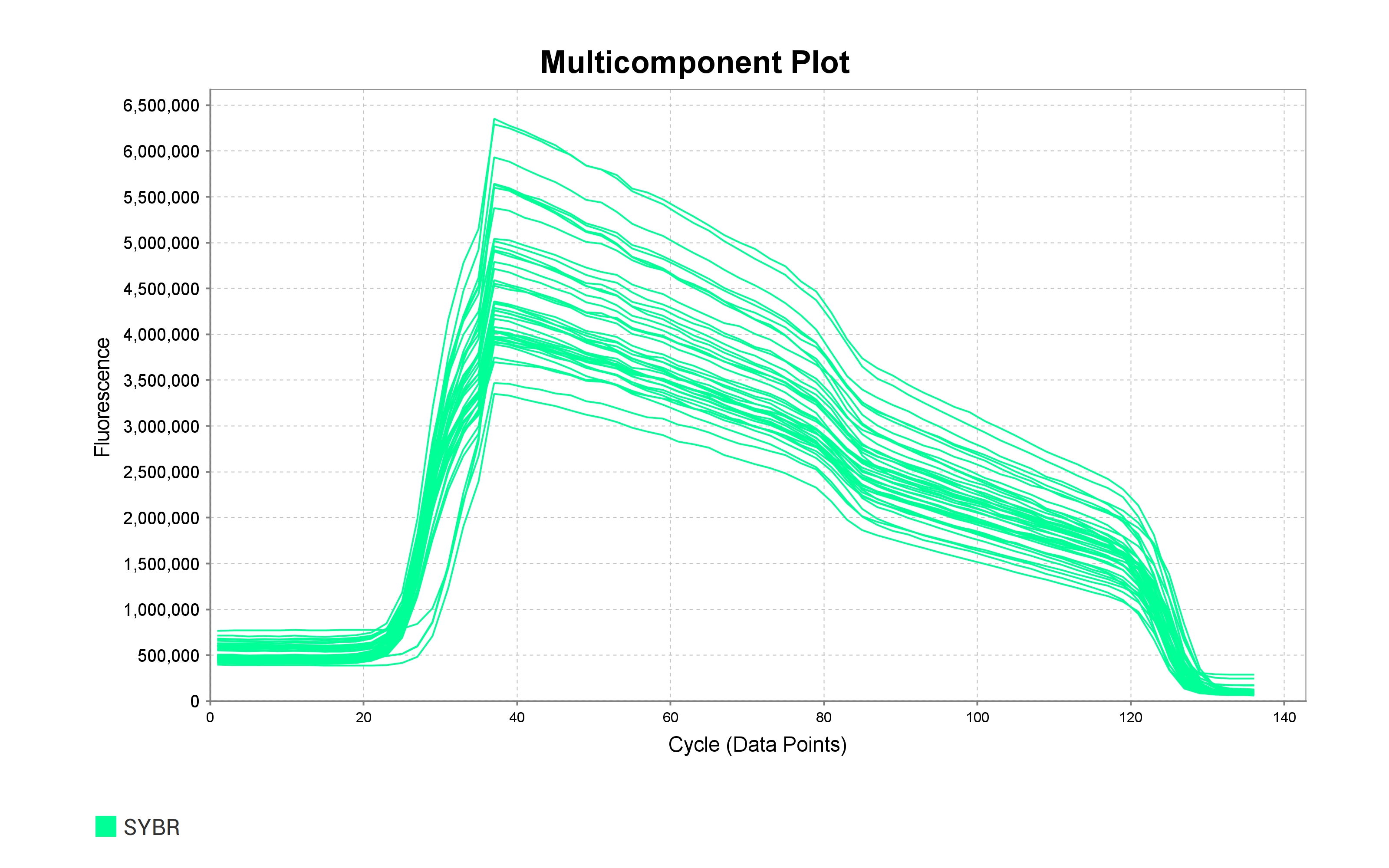

Supplement: Supplementary file 1 [file ijms-27-03895-s001.zip › pcrgraphs/Multicomponent Plot alb 27.2.24.jpg]

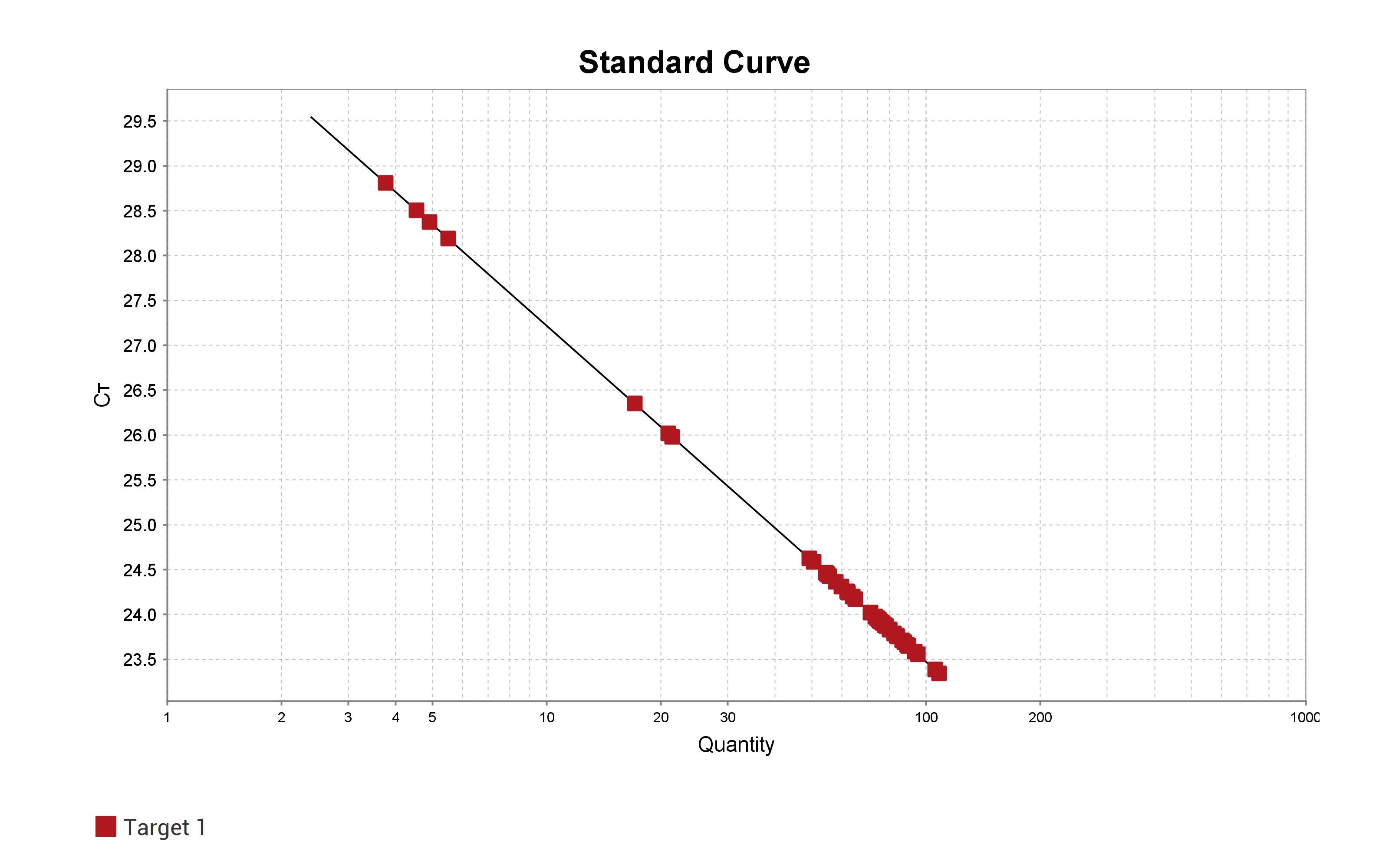

Supplement: Supplementary file 1 [file ijms-27-03895-s001.zip › pcrgraphs/Standard Curve 16.2.24 2nd.jpg]

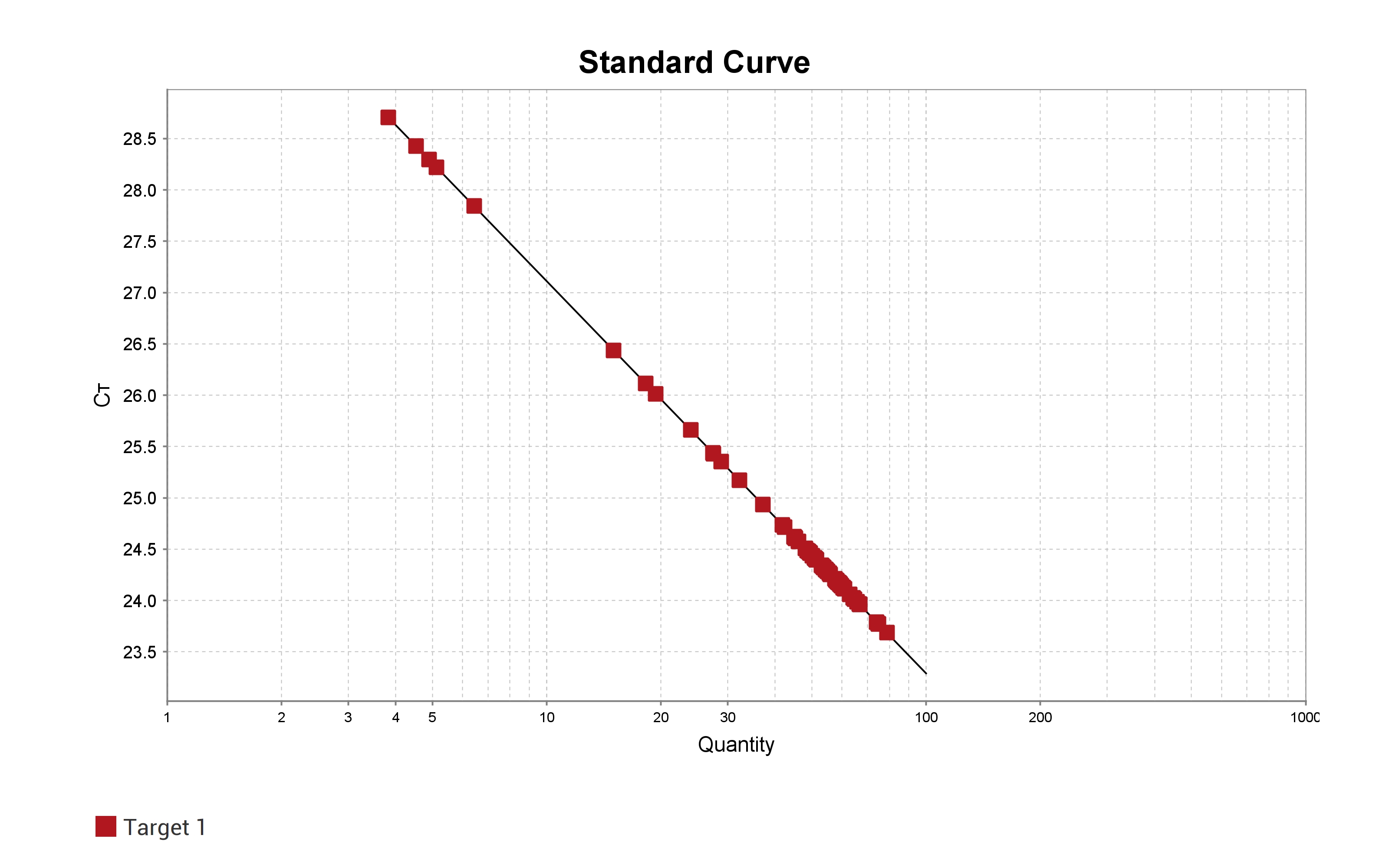

Supplement: Supplementary file 1 [file ijms-27-03895-s001.zip › pcrgraphs/Standard Curve Samples 13.12.23.jpg]

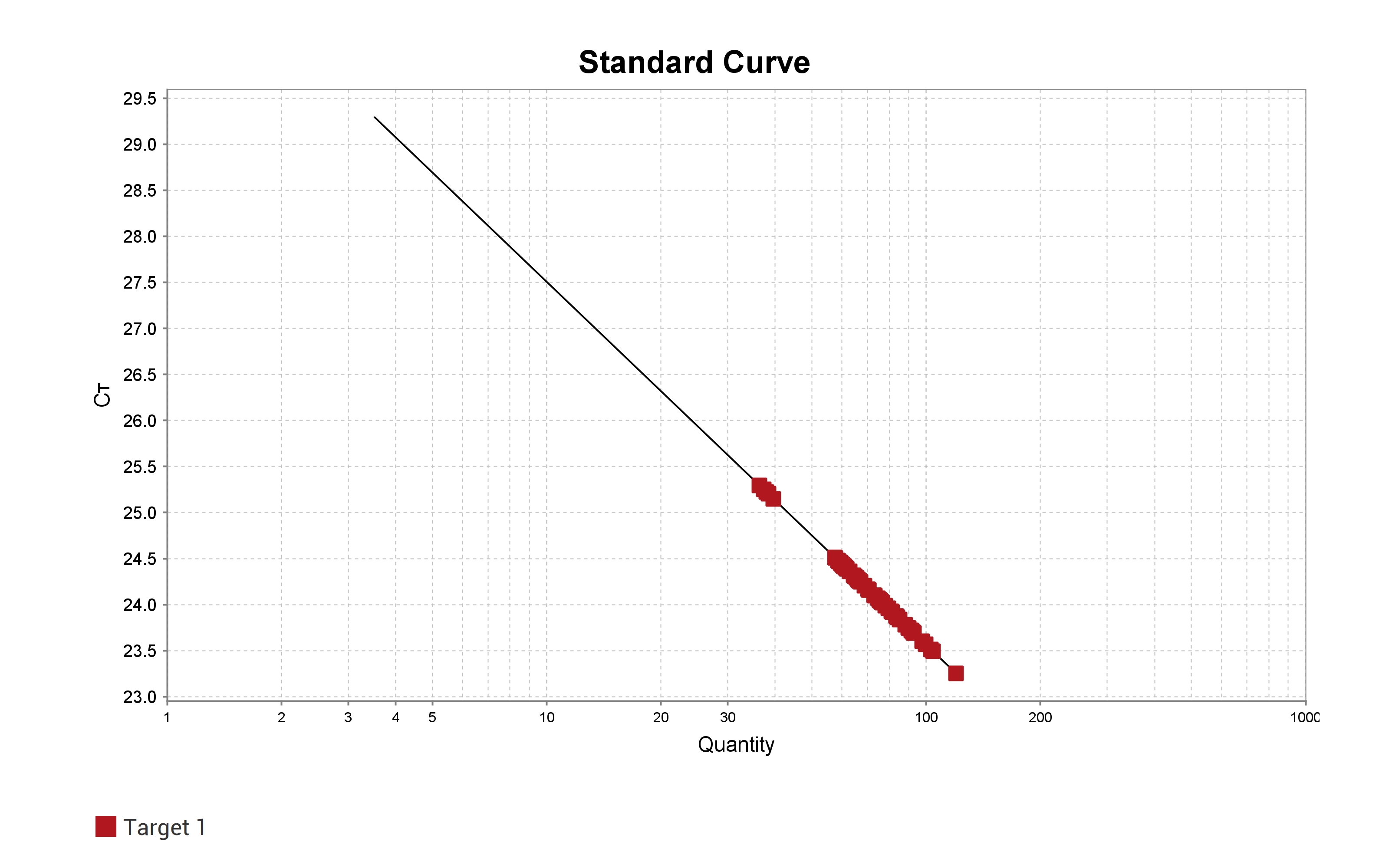

Supplement: Supplementary file 1 [file ijms-27-03895-s001.zip › pcrgraphs/Standard Curve Samples 16.2.24.jpg]
